# Spectral shift functions and Dirichlet-to-Neumann maps

**DOI:** 10.1007/s00208-017-1593-4

**Published:** 2017-09-23

**Authors:** Jussi Behrndt, Fritz Gesztesy, Shu Nakamura

**Affiliations:** 1 Institut für Numerische Mathematik, Technische Universität Graz, Steyrergasse 30, 8010 Graz, Austria; 2 Department of Mathematics, Baylor University, One Bear Place #97328, Waco, TX 76798-7328 USA; 3 Graduate School of Mathematical Sciences, University of Tokyo, 3-8-1, Komaba, Meguro-ku, Tokyo, 153-8914 Japan

**Keywords:** Primary 35J10, 35J15, 47A10, 47A40, 47B25, Secondary 35P20, 35P25, 47A55, 47B10, 47F05, 81Q10

## Abstract

The spectral shift function of a pair of self-adjoint operators is expressed via an abstract operator-valued Titchmarsh–Weyl *m*-function. This general result is applied to different self-adjoint realizations of second-order elliptic partial differential operators on smooth domains with compact boundaries and Schrödinger operators with compactly supported potentials. In these applications the spectral shift function is determined in an explicit form with the help of (energy parameter dependent) Dirichlet-to-Neumann maps.

## Introduction

Let *A* and *B* be self-adjoint operators in a separable Hilbert space ${\mathfrak H}$ and assume that the *m*-th powers of their resolvents differ by a trace class operator,

1.1 $$\begin{aligned} \big [(B- z I_{{\mathfrak H}})^{-m}-(A- z I_{{\mathfrak H}})^{-m}\big ]\in {\mathfrak S}_1({\mathfrak H}),\quad z\in \rho (A)\cap \rho (B), \end{aligned}$$

for some odd integer $m\in {\mathbb {N}}$. It is known that in this case there exists a real-valued function $\xi \in L^1_\mathrm{loc}({\mathbb {R}})$ such that $\int _{\mathbb {R}}\vert \xi (\lambda )\vert (1+\vert \lambda \vert )^{-(m+1)}d\lambda < \infty$ and the trace formula

1.2 $$\begin{aligned} {{\mathrm{tr}}}_{{\mathfrak H}}\bigl (\varphi (B)-\varphi (A)\bigr )=\int _{\mathbb {R}}\varphi ^\prime (\lambda ) \, \xi (\lambda )\,d\lambda \end{aligned}$$

holds for all suitable smooth functions $\varphi :{\mathbb {R}}\rightarrow {\mathbb {C}}$ such that $[\varphi (B)-\varphi (A)] \in {\mathfrak S}_1({\mathfrak H})$. The function $\xi$ in (1.2) is called a *spectral shift function* of the pair $\{A,B\}$. Note that for $\varphi (\lambda )=(\lambda - z)^{-m}$ one has $[\varphi (B)-\varphi (A)]\in {\mathfrak S}_1({\mathfrak H})$ according to (1.1) and the trace formula (1.2) takes the special form

$$\begin{aligned} {{\mathrm{tr}}}_{{\mathfrak H}}\bigl ((B- z I_{{\mathfrak H}})^{-m}-(A- z I_{{\mathfrak H}})^{-m}\bigr ) = -m \int _{\mathbb {R}}\frac{\xi (\lambda )\,d\lambda }{(\lambda - z)^{m+1}}. \end{aligned}$$

Historically the trace formula (1.2) was first proposed and verified on a formal level by Lifshitz for the case that $[B-A]$ is a finite-rank operator in [51] (see also [52]), and shortly afterwards in [44] Krein proved (1.2) rigorously in the more general case $[B-A]\in {\mathfrak S}_1({\mathfrak H})$ for all $C^1$-functions $\varphi$ with derivatives in the Wiener class. Furthermore, in [44] it was shown how the spectral shift function $\xi$ can be computed with the help of the perturbation determinant corresponding to the pair $\{A,B\}$. For pairs of unitary operators and thus via Cayley transforms for the case $m=1$ in (1.1) the spectral shift function and the trace formula were obtained later by Krein in [45]. Afterwards in [43] the more general case $m>1$ in (1.1) for self-adjoint operators *A* and *B* with $\rho (A)\cap \rho (B)\cap {\mathbb {R}}\not =\emptyset$ was discussed by Koplienko, and for odd integers *m* in (1.1) and arbitrary self-adjoint operators *A* and *B* see [74] by Yafaev or [73, Chapter 8, $\S$11] and [76, Chapter 0, Theorem 9.4]. We also mention that the spectral shift function is closely connected with the scattering matrix via the famous Birman–Krein formula from [11, 12]. For more details on the history, development and multifaceted applications of the spectral shift function in mathematical analysis we refer the reader to the survey papers [13, 16, 17], the standard monographs [73, 76], and, for instance, to [14, 19, 24, 26, 27, 31, 46, 47, 67, 70] and the more recent contributions [1, 25, 30, 39, 40, 42, 48, 55, 56, 64–66, 68, 75].

The main objective of the present paper is to prove a representation formula for the spectral shift function in terms of an abstract Titchmarsh–Weyl *m*-function of two self-adjoint operators satisfying the condition (1.1), and to apply this result to different self-adjoint realizations of second-order elliptic PDEs and Schrödinger operators with compactly supported potentials. In these applications the abstract Titchmarsh–Weyl *m*-function will turn out to be the energy dependent Neumann-to-Dirichlet map or Dirichlet-to-Neumann map associated to the elliptic differential expression and the Schrödinger operators on an interior and exterior domain, respectively.

More precisely, assume that *A* and *B* are self-adjoint operators in a separable Hilbert space ${\mathfrak H}$ and consider the underlying closed symmetric operator

$$\begin{aligned} Sf:=Af=Bf,\quad {{\mathrm{dom}}}(S):=\bigl \{f\in {{\mathrm{dom}}}(A)\cap {{\mathrm{dom}}}(B) \, \big | \, Af=Bf\bigr \}, \end{aligned}$$

which for convenience we assume is densely defined. We emphasize that neither *A* nor *B* needs to be semibounded in our approach. However, we first impose an implicit sign condition on the perturbation by assuming

1.3 $$\begin{aligned} (A-\mu _0 I_{{\mathfrak H}})^{-1}\ge (B-\mu _0 I_{{\mathfrak H}})^{-1} \end{aligned}$$

for some $\mu _0 \in \rho (A)\cap \rho (B)\cap {\mathbb {R}}$; in the semibounded case the condition (1.3) is equivalent to $A\le B$ interpreted in the sense of the corresponding quadratic forms. We then make use of the concept of quasi boundary triples in extension theory of symmetric operators from [2, 3] and construct an operator *T* such that $\overline{T}=S^*$ and two boundary mappings $\Gamma _0,\Gamma _1:{{\mathrm{dom}}}(T)\rightarrow {\mathcal {G}}$, where ${\mathcal {G}}$ is an auxiliary Hilbert space, such that

1.4 $$\begin{aligned} A=T\upharpoonright \ker (\Gamma _0)\quad \text { and } \quad B=T\upharpoonright \ker (\Gamma _1); \end{aligned}$$

see Proposition 2.4 and Sect. 2 for more details. To such a quasi boundary triple $\{{\mathcal {G}},\Gamma _0,\Gamma _1\}$ one associates the $\gamma$-field and Weyl function (or abstract Titchmarsh–Weyl *m*-function) *M* which are defined by

$$\begin{aligned} \gamma (z)\Gamma _0 f_z = f_z \quad \text { and }\quad M(z)\Gamma _0 f_z=\Gamma _1 f_z, \quad f_z \in \ker (T- z I_{{\mathfrak H}}), \; z\in \rho (A), \end{aligned}$$

respectively. Very roughly speaking the values *M*(*z*), $z \in \rho (A)$, of the function *M* map abstract Dirichlet boundary values to abstract Neumann boundary values, or vice versa, and hence the Weyl function *M* associated to a quasi boundary triple can be viewed as an abstract analog of the (energy parameter dependent) Dirichlet-to-Neumann map. The resolvents of *A* and *B* are related with the $\gamma$-field and Weyl function via the useful Krein-type formula

$$\begin{aligned} (B- z I_{{\mathfrak H}})^{-1}-(A- z I_{{\mathfrak H}})^{-1} = - \gamma (z) M(z)^{-1} \gamma ({\overline{z}})^*, \quad z\in \rho (A)\cap \rho (B). \end{aligned}$$

In our main result, Theorem 4.1, in the abstract part of this paper we provide sufficient ${\mathfrak S}_p$-type conditions on the $\gamma$-field and Weyl function of the quasi boundary triple $\{{\mathcal {G}},\Gamma _0,\Gamma _1\}$ such that (1.1) is satisfied with $m=2k+1$ and conclude that for any orthonormal basis $(\varphi _j)_{j \in J}$ in ${\mathcal {G}}$ (with $J \subseteq {\mathbb {N}}$ an appropriate index set), the function

1.5 $$\begin{aligned} \xi (\lambda )=\sum _{j \in J} \lim _{\varepsilon \downarrow 0}\pi ^{-1} \bigl (\mathrm {Im}\big (\log \big (\overline{M(\lambda +i\varepsilon )}\big )\big )\varphi _j,\varphi _j\bigr )_{\mathcal {G}}\quad \text { for a.e.}~\lambda \in {\mathbb {R}}, \end{aligned}$$

is a spectral shift function for the pair $\{A,B\}$ such that $\xi (\lambda )=0$ in an open neighborhood of $\mu _0$. In particular, the trace formula

$$\begin{aligned} {{\mathrm{tr}}}_{{\mathfrak H}}\bigl ( (B- z I_{{\mathfrak H}})^{-(2k+1)} - (A- z I_{{\mathfrak H}})^{-(2k+1)}\bigr ) = - (2k+1) \int _{\mathbb {R}}\frac{\xi (\lambda )\,d\lambda }{(\lambda - z)^{2k+2}} \end{aligned}$$

is valid for all $z \in \rho (A)\cap \rho (B)$. Furthermore, if (1.1) is satisfied with $m=1$ then according to Corollary 4.2 the imaginary part of the logarithm of $z \mapsto \overline{M(z)}$ is a trace class valued Nevanlinna (or Riesz–Herglotz) function on the open upper half-plane ${\mathbb {C}}_+$ (and hence admits nontangential limits for a.e. $\lambda \in {\mathbb {R}}$ from ${\mathbb {C}}_+$ in the trace class topology), and the spectral shift function in (1.5) has the form

1.6 $$\begin{aligned} \xi (\lambda )=\lim _{\varepsilon \downarrow 0}\pi ^{-1} {{\mathrm{tr}}}_{{\mathcal {G}}} \bigl (\mathrm {Im}\big (\log \big (\overline{M(\lambda +i\varepsilon )}\big )\big )\bigr ) \quad \text { for a.e.}~\lambda \in {\mathbb {R}}. \end{aligned}$$

Since $z \mapsto \log \big (\overline{M(z)}\big )$ is a Nevanlinna function it follows that the values of the spectral shift function $\xi$ in (1.5) and (1.6) are nonnegative for a.e. $\lambda \in {\mathbb {R}}$; this is rooted in the sign condition (1.3). In a second step we weaken the sign condition (1.3) and extend our representation of the spectral shift function to more general perturbations in the end of Sect. 4. We point out that the key difficulty in the proof of (1.5) and (1.6) is to ensure the existence of the limits on the right hand side of (1.5) and the trace class property of the function $\mathrm {Im}\big (\log \big (\overline{M}\big )\big )$ in the case $k=0$, respectively, which are indispensable for (1.5) and (1.6). These problems are investigated separately in Sect. 3 on the logarithm of operator-valued Nevanlinna functions, where special attention is paid to the analytic continuation by reflection with respect to open subsets of the real line. We also mention that for the special case where (1.1) is a rank one or finite-rank operator and $m=1$, our representation for the spectral shift function coincides with the one in [7, 49]. Furthermore, for $m=1$ in (1.1) a formula for the spectral shift function via a perturbation determinant involving boundary parameters and the Weyl function in the context of ordinary boundary triples was shown recently in [56] (see also [55]). We remark that our abstract result can also be formulated and remains valid in the special situation that the quasi boundary triple $\{{\mathcal {G}},\Gamma _0,\Gamma _1\}$ is a generalized or ordinary boundary triple in the sense of [18, 21–23, 32].

Our main reason to provide the general result in Sect. 4 for the spectral shift function in terms of the abstract notion of quasi boundary triples and their Weyl functions is its convenient applicability to various PDE situations, see also [2–6, 8] for other related applications of quasi boundary triples in PDE problems. In Sect. 5 we consider a formally symmetric uniformly elliptic second-order partial differential expression ${\mathcal {L}}$ with smooth coefficients on a bounded or unbounded domain in ${\mathbb {R}}^n$, $n\ge 2$, with compact boundary, and two self-adjoint realizations $A_{\beta _0}$ and $A_{\beta _1}$ of ${\mathcal {L}}$ subject to Robin boundary conditions $\beta _p\gamma _D f=\gamma _N f$, where $\gamma _D$ and $\gamma _N$ denote the Dirichlet and Neumann trace operators, and $\beta _p\in C^1(\partial \Omega )$, $p=0,1$, are real-valued functions. It then turns out that the Robin realizations $A_{\beta _0}$ and $A_{\beta _1}$ satisfy

1.7 $$\begin{aligned} \big [(A_{\beta _1}- z I_{L^2(\Omega )})^{-(2k+1)} - (A_{\beta _0}- z I_{L^2(\Omega )})^{-(2k+1)}\big ] \in {\mathfrak S}_1\bigl (L^2(\Omega )\bigr ) \end{aligned}$$

for all $k \in {\mathbb {N}}_0$, $k\ge (n-3)/4$, and $z \in \rho (A_{\beta _0})\cap \rho (A_{\beta _1})$, and for any orthonormal basis $(\varphi _j)_{j \in J}$ in $L^2(\partial \Omega )$, the function

1.8 $$\begin{aligned} \xi (\lambda )&=\sum _{j \in J} \lim _{\varepsilon \downarrow 0}\pi ^{-1} \Bigl (\bigl (\mathrm {Im}\bigl (\log ({\mathcal {M}}_1(\lambda +i\varepsilon ))-\log ({\mathcal {M}}_0(\lambda +i\varepsilon ))\bigr )\bigr )\varphi _j, \varphi _j\Bigr )_{L^2(\partial \Omega )}\nonumber \\&\quad \text {for a.e.}\,\lambda \in {\mathbb {R}}, \end{aligned}$$

is a spectral shift function for the pair $\{A_{\beta _0},A_{\beta _1}\}$, where

$$\begin{aligned} {\mathcal {M}}_p(z)= (\beta -\beta _p)^{-1}\bigl (\beta _p\overline{{\mathcal {N}}(z)}-I_{L^2(\partial \Omega )}\bigr )\bigl (\beta \overline{{\mathcal {N}}(z)}-I_{L^2(\partial \Omega )}\bigr )^{-1}, \quad z \in {\mathbb {C}}\backslash {\mathbb {R}}, \end{aligned}$$

$\beta \in {\mathbb {R}}$ is such that $\beta _p(x)<\beta$ for all $x\in \partial \Omega$, and ${\mathcal {N}}(z)$ denotes the (*z*-dependent) Neumann-to-Dirichlet map that assigns Neumann boundary values of solutions $f_z \in H^2(\Omega )$ of ${\mathcal {L}}f_z = z f_z$, $z \in {\mathbb {C}}\backslash {\mathbb {R}}$, onto their Dirichlet boundary values. We note that the trace class property (1.7) was shown in [4, 34] for the case $k=0$ and in [6] for $k\ge 1$. Moreover, in the case $k=0$, that is, $n=2$ or $n=3$, it follows from (1.6) that the spectral shift function in (1.8) has the form

$$\begin{aligned} \xi (\lambda )=\lim _{\varepsilon \downarrow 0}\pi ^{-1} {{\mathrm{tr}}}_{L^2(\partial \Omega )}\left( \mathrm {Im}\bigl (\log ({\mathcal {M}}_1(\lambda +i\varepsilon ))-\log ({\mathcal {M}}_0(\lambda +i\varepsilon ))\bigr )\right) \, \text { for a.e.}~\lambda \in {\mathbb {R}}. \end{aligned}$$

In our second example, presented in Sect. 6, we consider a Schrödinger operator $B = - \Delta +V$ with a compactly supported potential $V\in L^\infty ({\mathbb {R}}^n)$. Here we split the Euclidean space ${\mathbb {R}}^n$ and the Schrödinger operator via a multi-dimensional Glazman decomposition and consider the orthogonal sum $B_D=B_+\oplus C$ of the Dirichlet realizations of $-\Delta +V$ in $L^2({\mathcal {B}}_+)$ and $L^2({\mathcal {B}}_-)$, where ${\mathcal {B}}_+$ is a sufficiently large ball which contains $\text {supp}\,(V)$ and ${\mathcal {B}}_-:={\mathbb {R}}^n\backslash \overline{{\mathcal {B}}}_+$. Similarly, the unperturbed operator $A=-\Delta$ is decoupled and compared with the orthogonal sum $A_D=A_+\oplus C$ of the Dirichlet realizations of $-\Delta$ in $L^2({\mathcal {B}}_+)$ and $L^2({\mathcal {B}}_-)$. Our abstract result applies to the pairs $\{B,B_D\}$ and $\{A,A_D\}$, whenever $k> (n-2)/4$, $n \in {\mathbb {N}}$, $n \ge 2$, and yields an explicit formula for their spectral shift functions $\xi _B$ and $\xi _A$ in terms of the (*z*-dependent) Dirichlet-to-Neumann maps associated to $-\Delta$ and $-\Delta +V$ on ${\mathcal {B}}_+$ and ${\mathcal {B}}_-$. Since the spectra of the Dirichlet realizations $A_+=-\Delta$ and $B_+=-\Delta +V$ on the bounded domain ${\mathcal {B}}_+$ are both discrete and bounded from below, the difference of their eigenvalue counting functions is a spectral shift function $\xi _+$ for the pair $\{A_+,B_+\}$, and hence also for the pair $\{A_D,B_D\}$. Then it follows that the function

$$\begin{aligned} \xi (\lambda )=\xi _A(\lambda )-\xi _B(\lambda )+\xi _+(\lambda )\quad \text { for a.e.}~\lambda \in {\mathbb {R}}, \end{aligned}$$

is a spectral shift function for the original pair $\{A,B\}$ (cf. Theorem 6.1). We also mention that the trace class property of the resolvent differences of *A* and $A_D$, and *B* and $B_D$ goes back to Birman [9] and Grubb [33], and that similar decoupling methods are often used in scattering theory, see, for instance, [20] or [71] for a slighty more abstract and general framework.

The applications in Sects. 5 and 6 serve as typical examples for the abstract formalism and results in Sect. 4. In this context we mention that one may compare in a similar form as in Sect. 5 the Dirichlet realization with the Neumann, or other self-adjoint Robin realizations of an elliptic partial differential expression, and that in principle also higher-order differential expressions with smooth coefficients could be considered. We refer the reader to [28, 29, 35–37, 54, 57, 58, 63] for some recent related contributions in this area.

Finally, we briefly summarize the basic notation used in this paper: Let ${\mathcal {G}}$, ${\mathcal {H}}$, ${\mathfrak H}$, etc., be separable complex Hilbert spaces, $(\cdot ,\cdot )_{{\mathcal {H}}}$ the scalar product in ${\mathcal {H}}$ (linear in the first factor), and $I_{{\mathcal {H}}}$ the identity operator in ${\mathcal {H}}$. If *T* is a linear operator mapping (a subspace of ) a Hilbert space into another, ${{\mathrm{dom}}}(T)$ denotes the domain and ${{\mathrm{ran}}}(T)$ is the range of *T*. The closure of a closable operator *S* is denoted by $\overline{S}$. The spectrum and resolvent set of a closed linear operator in ${\mathcal {H}}$ will be denoted by $\sigma (\cdot )$ and $\rho (\cdot )$, respectively. The Banach space of bounded linear operators in ${\mathcal {H}}$ is denoted by ${\mathcal {L}}({\mathcal {H}})$; in the context of two Hilbert spaces, ${\mathcal {H}}_j$, $j=1,2$, we use the analogous abbreviation ${\mathcal {L}}({\mathcal {H}}_1, {\mathcal {H}}_2)$. The *p*-th Schatten–von Neumann ideal consists of compact operators with singular values in $l^p$, $p>0$, and is denoted by ${\mathfrak S}_p({\mathcal {H}})$ and ${\mathfrak S}_p({\mathcal {H}}_1,{\mathcal {H}}_2)$. For $\Omega \subseteq {\mathbb {R}}^n$ nonempty, $n \in {\mathbb {N}}$, we suppress the *n*-dimensional Lebesgue measure $d^n x$ and use the shorthand notation $L^2(\Omega ) := L^2(\Omega ; d^n x)$; similarly, if $\partial \Omega$ is sufficiently regular we write $L^2(\partial \Omega ) := L^2(\partial \Omega ; d^{n-1} \sigma )$, with $d^{n-1} \sigma$ the surface measure on $\partial \Omega$. We also abbreviate ${\mathbb {C}}_{\pm } := \{z \in {\mathbb {C}}\, | \, \mathrm {Im}(z) \gtrless 0\}$ and ${\mathbb {N}}_0 = {\mathbb {N}}\cup \{0\}$.

## Quasi boundary triples and their Weyl functions

In this section we recall the concept of quasi boundary triples and their Weyl functions from extension theory of symmetric operators. We shall make use of these notions in Sect. 4 and formulate our main abstract result Theorem 4.1 in terms of the Weyl function of a quasi boundary triple. In Sects. 5 and 6 quasi boundary triples and their Weyl functions are used to parametrize self-adjoint Schrödinger operators and self-adjoint elliptic differential operators with suitable boundary conditions. We refer to [2, 3] for more details on quasi boundary triples and to [4–6, 8] for some applications; for the related notions of generalized and ordinary boundary triples see [18, 21–23, 32, 69].

Throughout this section let ${\mathfrak H}$ be a separable Hilbert space and let *S* be a densely defined closed symmetric operator in ${\mathfrak H}$.

### Definition 2.1

Let $T\subset S^*$ be a linear operator in ${\mathfrak H}$ such that $\overline{T}=S^*$. A triple $\{{\mathcal {G}},\Gamma _0,\Gamma _1\}$ is said to be a *quasi boundary triple* for $T\subset S^*$ if ${\mathcal {G}}$ is a Hilbert space and $\Gamma _0,\Gamma _1:{{\mathrm{dom}}}(T)\rightarrow {\mathcal {G}}$ are linear mappings such that the following conditions (i)–(iii) are satisfied:

(i) The abstract Green’s identity
  $$\begin{aligned} (Tf,g)_{\mathfrak H}-(f,Tg)_{\mathfrak H}=(\Gamma _1 f,\Gamma _0 g)_{\mathcal {G}}-(\Gamma _0 f,\Gamma _1 g)_{\mathcal {G}}\end{aligned}$$
  holds for all $f,g\in {{\mathrm{dom}}}(T)$.
(ii) The range of the map $(\Gamma _0,\Gamma _1)^\top :{{\mathrm{dom}}}(T)\rightarrow {\mathcal {G}}\times {\mathcal {G}}$ is dense.
(iii) The operator $A_0:=T\upharpoonright \ker (\Gamma _0)$ is self-adjoint in ${\mathfrak H}$.

The next theorem from [2, 3] is useful in the applications in Sects. 5 and 6; it contains a sufficient condition for a triple $\{{\mathcal {G}},\Gamma _0,\Gamma _1\}$ to be a quasi boundary triple.

### Theorem 2.2

Let ${\mathfrak H}$ and ${\mathcal {G}}$ be separable Hilbert spaces and let *T* be a linear operator in ${\mathfrak H}$. Assume that $\Gamma _0,\Gamma _1: {{\mathrm{dom}}}(T)\rightarrow {\mathcal {G}}$ are linear mappings such that the following conditions (i)–(iii) hold:

(i) The abstract Green’s identity
  $$\begin{aligned} (Tf,g)_{\mathfrak H}-(f,Tg)_{\mathfrak H}=(\Gamma _1 f,\Gamma _0 g)_{\mathcal {G}}-(\Gamma _0 f,\Gamma _1 g)_{\mathcal {G}}\end{aligned}$$
  holds for all $f,g\in {{\mathrm{dom}}}(T)$.
(ii) The range of $(\Gamma _0,\Gamma _1)^\top : {{\mathrm{dom}}}(T)\rightarrow {\mathcal {G}}\times {\mathcal {G}}$ is dense and $\ker (\Gamma _0)\cap \ker (\Gamma _1)$ is dense in ${\mathfrak H}$.
(iii) $T\upharpoonright \ker (\Gamma _0)$ is an extension of a self-adjoint operator $A_0$.

Then

$$\begin{aligned} S:= T\upharpoonright \bigl (\ker (\Gamma _0)\cap \ker (\Gamma _1)\bigr ) \end{aligned}$$

is a densely defined closed symmetric operator in ${\mathfrak H}$ such that $\overline{T}= S^*$ holds and the triple $\{{\mathcal {G}},\Gamma _0,\Gamma _1\}$ is a quasi boundary triple for $S^*$ with $A_0=T\upharpoonright \ker (\Gamma _0)$.

Next, we recall the definition of the $\gamma$-field $\gamma$ and Weyl function *M* associated to a quasi boundary triple, which is formally the same as in [22, 23] for the case of ordinary or generalized boundary triples. Let $\{{\mathcal {G}},\Gamma _0,\Gamma _1\}$ be a quasi boundary triple for $T\subset S^*$ with $A_0=T\upharpoonright \ker (\Gamma _0)$ and note that the direct sum decomposition

2.1 $$\begin{aligned} {{\mathrm{dom}}}(T) = {{\mathrm{dom}}}(A_0)\,\dot{+}\,\ker (T - z I_{{\mathfrak H}}) = \ker (\Gamma _0)\,\dot{+}\,\ker (T- z I_{{\mathfrak H}}) \end{aligned}$$

of ${{\mathrm{dom}}}(T)$ holds for all $z \in \rho (A_0)$. Hence the mapping $\Gamma _0\upharpoonright \ker (T - z I_{{\mathfrak H}})$ is injective for all $z \in \rho (A_0)$ and its range coincides with ${{\mathrm{ran}}}(\Gamma _0)$.

### Definition 2.3

Let $T\subset S^*$ be a linear operator in ${\mathfrak H}$ such that $\overline{T}=S^*$ and let $\{{\mathcal {G}},\Gamma _0,\Gamma _1\}$ be a quasi boundary triple for $T\subset S^*$ with $A_0=T\upharpoonright \ker (\Gamma _0)$. The $\gamma$ *-field* $\gamma$ and the *Weyl function* *M* corresponding to $\{{\mathcal {G}},\Gamma _0,\Gamma _1\}$ are operator-valued functions on $\rho (A_0)$ which are defined by

$$\begin{aligned} z \mapsto \gamma (z):=\bigl (\Gamma _0\upharpoonright \ker (T - z I_{{\mathfrak H}})\bigr )^{-1} \, \text { and } \, z \mapsto M(z) := \Gamma _1\bigl (\Gamma _0\upharpoonright \ker (T - z I_{{\mathfrak H}})\bigr )^{-1}. \end{aligned}$$

Various properties of the $\gamma$-field and Weyl function were provided in [2, 3], see also [18, 21–23, 69] for the special cases of ordinary and generalized boundary triples. We briefly review some items which are important for our purposes. Note first that the values $\gamma (z)$, $z \in \rho (A_0)$, of the $\gamma$-field are operators defined on the dense subspace ${{\mathrm{ran}}}(\Gamma _0)\subset {\mathcal {G}}$ which map onto $\ker (T - z I_{{\mathfrak H}})\subset {\mathfrak H}$. The operators $\gamma (z)$, $z \in \rho (A_0)$, are bounded and admit continuous extensions $\overline{\gamma (z)}\in {\mathcal {L}}({\mathcal {G}},{\mathfrak H})$. For the adjoint operators $\gamma (z)^*\in {\mathcal {L}}({\mathfrak H},{\mathcal {G}})$, $z \in \rho (A_0)$, it follows that

2.2 $$\begin{aligned} \gamma (z)^*=\Gamma _1(A_0-{\overline{z}} I_{{\mathfrak H}})^{-1},\quad z \in \rho (A_0), \end{aligned}$$

and, in particular, ${{\mathrm{ran}}}(\gamma (z)^*)={{\mathrm{ran}}}(\Gamma _1\upharpoonright {{\mathrm{dom}}}(A_0))$ does not depend on $z \in \rho (A_0)$. It is also important to note that $({{\mathrm{ran}}}(\gamma (z)^*))^\bot =\ker (\gamma (z))=\{0\}$ and hence

2.3 $$\begin{aligned} \overline{{{\mathrm{ran}}}(\gamma (z)^*)}={\mathcal {G}},\quad z \in \rho (A_0). \end{aligned}$$

In the same way as for ordinary boundary triples one verifies

2.4 $$\begin{aligned} \gamma (z)\varphi =\bigl (I_{\mathfrak H}+(z - z_0)(A_0 - z I_{{\mathfrak H}})^{-1}\bigr )\gamma (z_0)\varphi , \quad z, z_0 \in \rho (A_0),\quad \varphi \in {{\mathrm{ran}}}(\Gamma _0), \end{aligned}$$

and therefore $z \mapsto \gamma (z)\varphi$ is holomorphic on $\rho (A_0)$ for all $\varphi \in {{\mathrm{ran}}}(\Gamma _0)$. The relation (2.4) extends by continuity to

2.5 $$\begin{aligned} \overline{\gamma (z)}=\bigl (I_{\mathfrak H}+(z - z_0)(A_0 - z I_{{\mathfrak H}})^{-1}\bigr )\overline{\gamma (z_0)} \in {\mathcal {L}}({\mathcal {G}},{\mathfrak H}),\quad z, z_0 \in \rho (A_0), \end{aligned}$$

and it follows that $z \mapsto \overline{\gamma (z)}$ is a holomorphic ${\mathcal {L}}({\mathcal {G}},{\mathfrak H})$-valued operator function. According to [6, Lemma 2.4] the identities

2.6 $$\begin{aligned} \frac{d^k}{dz^k}\overline{\gamma (z)}=k! \, (A_0 - z I_{{\mathfrak H}})^{-k}\overline{\gamma (z)}, \quad \frac{d^k}{dz^k}\gamma ({\overline{z}})^*=k! \, \gamma ({\overline{z}})^*(A_0 - z I_{{\mathfrak H}})^{-k}, \end{aligned}$$

hold for all $k \in {\mathbb {N}}_0$ and $z \in \rho (A_0)$.

The values *M*(*z*), $z \in \rho (A_0)$, of the Weyl function *M* associated to a quasi boundary triple are operators in ${\mathcal {G}}$ and it follows from Definition 2.3 that

$$\begin{aligned} {{\mathrm{dom}}}(M(z)) = {{\mathrm{ran}}}(\Gamma _0) \quad \text{ and } \quad {{\mathrm{ran}}}(M(z)) \subset {{\mathrm{ran}}}(\Gamma _1) \end{aligned}$$

hold for all $z \in \rho (A_0)$. In particular, the operators *M*(*z*), $z \in \rho (A_0)$, are densely defined in ${\mathcal {G}}$. With the help of the abstract Green’s identity one concludes that for $z, z_0 \in \rho (A_0)$ and $\varphi ,\psi \in {{\mathrm{ran}}}(\Gamma _0)$ the Weyl function and the $\gamma$-field satisfy

2.7 $$\begin{aligned} (M(z)\varphi ,\psi )_{\mathcal {G}}-(\varphi ,M(z_0)\psi )_{\mathcal {G}}=(z - \overline{z_0})\bigl (\gamma (z)\varphi ,\gamma (z_0)\psi \bigr )_{\mathcal {G}}\end{aligned}$$

and hence $M(z)\subset M({\overline{z}})^*$ and the operators *M*(*z*) are closable for all $z\in \rho (A_0)$. From (2.7) it also follows that the Weyl function and the $\gamma$-field are connected via

2.8 $$\begin{aligned} M(z)\varphi - M(z_0)^*\varphi =(z - \overline{z_0})\gamma (z_0)^*\gamma (z)\varphi , \quad z, z_0 \in \rho (A_0), \quad \varphi \in {{\mathrm{ran}}}(\Gamma _0). \end{aligned}$$

From (2.8) and (2.4) one obtains

2.9 $$\begin{aligned} \mathrm {Im}(M(z))\varphi = \mathrm {Im}(z)\,\gamma (z)^*\gamma (z)\varphi ,\quad z \in \rho (A_0), \quad \varphi \in {{\mathrm{ran}}}(\Gamma _0), \end{aligned}$$

and

2.10 $$\begin{aligned} M(z)\varphi&=\mathrm {Re}(M(z_0))\varphi \nonumber \\&\quad +\gamma (z_0)^*\bigl ((z - \mathrm {Re}(z_0))+(z - z_0)(z - \overline{z_0})(A_0 - z I_{{\mathfrak H}})^{-1}\bigr )\gamma (z_0)\varphi \end{aligned}$$

for all $z,z_0\in \rho (A_0)$ and $\varphi \in {{\mathrm{ran}}}(\Gamma _0)$. One observes that $z \mapsto M(z)\varphi$ is holomorphic on $\rho (A_0)$ for all $\varphi \in {{\mathrm{ran}}}(\Gamma _0)$ and by (2.9) the imaginary part of *M*(*z*) is a bounded operator in ${\mathcal {G}}$ which admits a bounded continuation to

2.11 $$\begin{aligned} \overline{\mathrm {Im}(M(z))} = \mathrm {Im}(z)\,\gamma (z)^*\overline{\gamma (z)}\in {\mathcal {L}}({\mathcal {G}}). \end{aligned}$$

Furthermore, the derivatives $\frac{d^k}{dz^k} M(z)$, $k \in {\mathbb {N}}$, of the Weyl function are densely defined bounded operators in ${\mathcal {G}}$ and according to [6, Lemma 2.4] one has

$$\begin{aligned} \overline{\frac{d^k}{dz^k} M(z)}=k! \, \gamma ({\overline{z}})^*(A_0 - z I_{{\mathfrak H}})^{-(k-1)}\overline{\gamma (z)}, \quad k \in {\mathbb {N}}, \; z \in \rho (A_0). \end{aligned}$$

If the values *M*(*z*) are densely defined bounded operators for some, and hence for all $z \in \rho (A_0)$ then

2.12 $$\begin{aligned} \frac{d^k}{dz^k}\overline{M(z)}=k! \, \gamma ({\overline{z}})^*(A_0 - z I_{{\mathfrak H}})^{-(k-1)}\overline{\gamma (z)}, \quad k \in {\mathbb {N}}, \quad z \in \rho (A_0). \end{aligned}$$

The next result will be used in the formulation and proof of our abstract representation formula for the spectral shift function in Sect. 4. The existence of a quasi boundary triple follows from [8, Proposition 2.9(i)] and the Krein-type resolvent formula in (2.14) is a special case of [3, Corollary 6.17] or [5, Corollary 3.9].

### Proposition 2.4

Let *A* and *B* be self-adjoint operators in ${\mathfrak H}$ and assume that the closed symmetric operator $S=A\cap B$ is densely defined. Then the closure of the operator

$$T=S^*\upharpoonright ({{\mathrm{dom}}}(A)+{{\mathrm{dom}}}(B))$$

coincides with $S^*$ and there exists a quasi boundary triple $\{{\mathcal {G}},\Gamma _0,\Gamma _1\}$ for $T\subset S^*$ such that

2.13 $$\begin{aligned} A=T\upharpoonright \ker (\Gamma _0)\quad \text { and } \quad B=T\upharpoonright \ker (\Gamma _1). \end{aligned}$$

Furthermore, if $\gamma$ and *M* are the corresponding $\gamma$-field and Weyl function then

2.14 $$\begin{aligned} (B - z I_{{\mathfrak H}})^{-1}-(A - z I_{{\mathfrak H}})^{-1}=-\gamma (z) M(z)^{-1}\gamma ({\overline{z}})^*, \quad z \in \rho (A)\cap \rho (B). \end{aligned}$$

## Logarithms of operator-valued Nevanlinna functions

In this section we study the logarithm of operator-valued Nevanlinna (or Nevanlinna–Herglotz, resp., Riesz–Herglotz) functions. Here we shall recall some of the results formulated in [26, Section 2] which go back to [10, 60–62], and slightly extend and reformulate these in a form convenient for our subsequent purposes.

We first recall the integral representation of the logarithm that corresponds to the cut along the negative imaginary axis,

3.1 $$\begin{aligned} \log (z)=-i\int _0^\infty \left( \frac{1}{z+i \lambda }-\frac{1}{1+i \lambda }\right) d\lambda , \quad z\in {\mathbb {C}},\;\; z\not =-i \lambda ,\;\; \lambda \ge 0. \end{aligned}$$

Next, let ${\mathcal {G}}$ be a separable Hilbert space and let $K\in {\mathcal {L}}({\mathcal {G}})$ be a bounded operator such that $\mathrm {Im}(K) \ge 0$ and $0\subset \rho (K)$. We use

3.2 $$\begin{aligned} \log (K):=-i\int _0^\infty \bigl [(K+ i \lambda I_{{\mathcal {G}}})^{-1}-(1+ i \lambda )^{-1}I_{\mathcal {G}}\bigr ] \, d\lambda \end{aligned}$$

as the definition of the logarithm of the operator *K*. Then $\log (K)\in {\mathcal {L}}({\mathcal {G}})$ by [26, Lemma 2.6] and in the special case that $K\in {\mathcal {L}}({\mathcal {G}})$ is self-adjoint and $0\in \rho (K)$, it follows from [26, Lemma 2.7] that

3.3 $$\begin{aligned} \mathrm {Im}(\log (K))=\pi E_K((-\infty ,0)), \end{aligned}$$

where $E_K(\cdot )$ is the spectral measure of *K*. In particular, if $K\in {\mathcal {L}}({\mathcal {G}})$ is self-adjoint and $0\in \rho (K)$ then $\sigma (K)\subset (0,\infty )$ if and only if $\log (K)$ is a self-adjoint operator.

In the next lemma we show that besides $\log (K)$ also $\log (K^*)$ is well-defined via (3.2) when *K* is a dissipative operator with spectrum off the imaginary axis (cf. [26, Lemmas 2.6, 2.7]).

### Lemma 3.1

Let $K\in {\mathcal {L}}({\mathcal {G}})$ be a dissipative operator such that $i \lambda \in \rho (K)$ for all $\lambda \ge 0$, and define

3.4 $$\begin{aligned} \log (K^*):=-i\int _0^\infty \bigl [(K^*+ i \lambda I_{{\mathcal {G}}})^{-1}-(1+ i \lambda )^{-1}I_{\mathcal {G}}\bigr ] \, d\lambda . \end{aligned}$$

Then $$\log (K^*)\in {\mathcal {L}}({\mathcal {G}})$$.

### Proof

From $\sigma (K^*)=\{z\in {\mathbb {C}}\, | \, {\overline{z}}\in \sigma (K)\}$ and the assumption $i\lambda \in \rho (K)$ for $\lambda \ge 0$ it is clear that $- i \lambda \in \rho (K^*)$ for $\lambda \ge 0$. Since *K* is dissipative it follows that $K^*$ is accretive, that is, $\mathrm {Im}(K^*) \le 0$. For $\delta >0$ one estimates

3.5 $$\begin{aligned} \begin{aligned} \Vert \log (K^*)\Vert _{{\mathcal {L}}({\mathcal {G}})}&\le \int _0^\delta \bigl [\big \Vert (K^*+ i \lambda I_{{\mathcal {G}}})^{-1}\big \Vert _{{\mathcal {L}}({\mathcal {G}})} + 1\bigr ] \, d\lambda \\&\quad + \int _\delta ^\infty \big \Vert (K^*+ i \lambda I_{{\mathcal {G}}})^{-1}\big \Vert _{{\mathcal {L}}({\mathcal {G}})} \bigl (\Vert K\Vert _{{\mathcal {L}}({\mathcal {G}})} + 1\bigr ) \lambda ^{-1}\, d\lambda . \end{aligned} \end{aligned}$$

For $0< \lambda < \big \Vert (K^*)^{-1}\big \Vert ^{-1}_{{\mathcal {L}}({\mathcal {G}})}$ one has

$$\begin{aligned} \big \Vert (K^*+ i \lambda I_{{\mathcal {G}}})^{-1}\big \Vert _{{\mathcal {L}}({\mathcal {G}})} \le \frac{\big \Vert (K^*)^{-1}\big \Vert _{{\mathcal {L}}({\mathcal {G}})}}{1-\lambda \big \Vert (K^*)^{-1}\big \Vert _{{\mathcal {L}}({\mathcal {G}})}}, \end{aligned}$$

and with the choice $\delta = \big (2 \big \Vert (K^*)^{-1}\big \Vert _{{\mathcal {L}}({\mathcal {G}})}\big )^{-1}$ it follows that the first integral in (3.5) is bounded. In order to show that the second integral in (3.5) is also bounded it suffices to show that

3.6 $$\begin{aligned} \big \Vert (K^*+ i \lambda I_{{\mathcal {G}}})^{-1}\big \Vert _{{\mathcal {L}}({\mathcal {G}})} \le \frac{1}{\lambda - \Vert K^*\Vert _{{\mathcal {L}}({\mathcal {G}})}}, \quad \lambda > \Vert K^*\Vert _{{\mathcal {L}}({\mathcal {G}})}. \end{aligned}$$

In fact, since $\mathrm {Im}(K^*+i\Vert K^*\Vert _{{\mathcal {L}}({\mathcal {G}})} I_{{\mathcal {G}}})\ge 0$ one estimates for $\lambda > \Vert K^*\Vert _{{\mathcal {L}}({\mathcal {G}})}$,

3.7 $$\begin{aligned} \begin{aligned} 0&\le (\lambda - \Vert K^*\Vert _{{\mathcal {L}}({\mathcal {G}})})\Vert f\Vert ^2_{{\mathcal {G}}} =\mathrm {Im}\bigl (i(\lambda - \Vert K^*\Vert _{{\mathcal {L}}({\mathcal {G}})}) f,f\bigr )_{{\mathcal {G}}} \\&\le \mathrm {Im}\bigl ((i \lambda - i\Vert K^*\Vert _{{\mathcal {L}}({\mathcal {G}})}) f,f\bigr )_{{\mathcal {G}}} +\mathrm {Im}\bigl ((K^*+i\Vert K^*\Vert _{{\mathcal {L}}({\mathcal {G}})} I_{{\mathcal {G}}}) f,f\bigr )_{{\mathcal {G}}} \\&=\mathrm {Im}\bigl ((K^*+ i \lambda I_{{\mathcal {G}}})f,f\bigr )_{{\mathcal {G}}} \le \Vert (K^*+ i \lambda I_{{\mathcal {G}}})f\Vert _{{\mathcal {G}}} \Vert f\Vert _{{\mathcal {G}}} \end{aligned} \end{aligned}$$

and for $f\not =0$ this yields

3.8 $$\begin{aligned} 0\le (\lambda - \Vert K^*\Vert _{{\mathcal {L}}({\mathcal {G}})})\Vert f\Vert _{{\mathcal {G}}} \le \Vert (K^*+ i \lambda I_{{\mathcal {G}}})f\Vert _{{\mathcal {G}}}. \end{aligned}$$

Since $- i \lambda \in \rho (K^*)$ there exists $g\in {\mathcal {G}}$ such that $f=(K^*+ i \lambda I_{{\mathcal {G}}})^{-1}g$ and then (3.8) has the form

$$\begin{aligned} \big \Vert (K^*+ i \lambda I_{{\mathcal {G}}})^{-1} g \big \Vert _{{\mathcal {G}}} \le \frac{1}{\lambda - \Vert K^*\Vert _{{\mathcal {L}}({\mathcal {G}})}} \Vert g\Vert _{{\mathcal {G}}}, \quad \lambda > \Vert K^*\Vert _{{\mathcal {L}}({\mathcal {G}})}. \end{aligned}$$

This implies (3.6), and hence the second integral in the estimate (3.5) is finite. Thus, $\log (K^*)$ in (3.4) is a bounded operator in ${\mathcal {G}}$. $\square$

We recall that a function $N:{\mathbb {C}}_+\rightarrow {\mathcal {L}}({\mathcal {G}})$ is an operator-valued *Nevanlinna* (or *Riesz–Herglotz*) *function* if *N* is holomorphic and $\mathrm {Im}(N(z))\ge 0$ holds for all $z\in {\mathbb {C}}_+$. An ${\mathcal {L}}({\mathcal {G}})$-valued Nevanlinna function is extended onto ${\mathbb {C}}_-$ by setting

3.9 $$\begin{aligned} N(z):=N({\overline{z}})^*,\quad z\in {\mathbb {C}}_-. \end{aligned}$$

We shall say that a Nevanlinna function *N* admits an *analytic continuation by reflection* with respect to some open subset $I\subset {\mathbb {R}}$ if *N* can be continued analytically from ${\mathbb {C}}_+$ onto an open set ${\mathcal {O}}\subset {\mathbb {C}}$ which contains *I* such that the values of the continuation in ${\mathcal {O}}\cap {\mathbb {C}}_-$ coincide with the values of *N* in (3.9) there.

### Example 3.2

If $\sqrt{z}$ is fixed for ${\mathbb {C}}\backslash [0,\infty )$ by $\mathrm {Im}(\sqrt{z}) > 0$ and by $\sqrt{z}\ge 0$ for $z\in [0,\infty )$ then ${\mathbb {C}}_+\ni z\mapsto \sqrt{z}$ is a (scalar ) Nevanlinna function which admits an analytic continuation by reflection with respect to $(-\infty ,0)$, but it does not admit an analytic continuation by reflection with respect to any open subinterval of $[0,\infty )$.

An operator-valued Nevanlinna function admits a minimal operator representation via the resolvent of a self-adjoint operator or relation in an auxiliary or larger Hilbert space (see, e.g., [10, 38, 50, 60]). More precisely, if $N:{\mathbb {C}}_+\rightarrow {\mathcal {L}}({\mathcal {G}})$ is a Nevanlinna function and $z_0\in {\mathbb {C}}_+$ is fixed then there exists a Hilbert space ${\mathcal {K}}$, a self-adjoint operator or self-adjoint relation *L* in ${\mathcal {K}}$ and an operator $R \in {\mathcal {L}}({\mathcal {G}},{\mathcal {K}})$ (depending on the choice of $z_0$) such that

3.10 $$\begin{aligned} N(z) = \mathrm {Re}(N(z_0)) + (z - \mathrm {Re}(z_0)) R^* R + (z - z_0)(z - {\overline{z}}_0)R^*(L - z I_{{\mathcal {K}}})^{-1}R\quad \end{aligned}$$

holds for $z \in {\mathbb {C}}_+$. If *N* satisfies the condition

3.11 $$\begin{aligned} \lim _{y\uparrow +\infty } y^{-1}(N(iy)h,h)_{{\mathcal {G}}}=0\, \text { for all } \, h\in {\mathcal {G}}, \end{aligned}$$

then *L* in (3.10) is a self-adjoint operator in ${\mathcal {K}}$; cf. [50, Corollary 2.5]. The representation (3.10) also holds for $z\in {\mathbb {C}}_-$ when *N* is extended onto ${\mathbb {C}}_-$ via (3.9). Note that the model can be chosen *minimal*, that is, the minimality condition

$$\begin{aligned} {\mathcal {K}}=\text {clsp}\,\bigl \{(I_{\mathcal {K}}+(z - z_0)(L - z I_{{\mathcal {K}}})^{-1})R h \, \big | \, z\in {\mathbb {C}}\backslash {\mathbb {R}}, \, h\in {\mathcal {G}}\bigr \} \end{aligned}$$

is satisfied, in which case the resolvent set $\rho (L)$ of *L* coincides with the maximal domain of analyticity of the function *N*. In particular, in this case *N* admits an analytic continuation by reflection with respect to an open subset $I\subset {\mathbb {R}}$ if and only if $I\subset \rho (L)$, and the open subset $\rho (L)\cap {\mathbb {R}}$ is maximal with this property.

Next, assume that *N* is an ${\mathcal {L}}({\mathcal {G}})$-valued Nevanlinna function and suppose that $N(z)^{-1}\in {\mathcal {L}}({\mathcal {G}})$ for some, and hence (by [26, Lemma 2.3]) for all $z\in {\mathbb {C}}\backslash {\mathbb {R}}$. Then we define for $z\in {\mathbb {C}}_+$ the logarithm $\log (N(z))$ in accordance with (3.2) by

3.12 $$\begin{aligned} \log (N(z)):=-i\int _0^\infty \bigl [(N(z)+ i \lambda I_{{\mathcal {G}}})^{-1}-(1+ i \lambda )^{-1}I_{\mathcal {G}}\bigr ] \, d\lambda , \end{aligned}$$

and extend the function $\log (N)$ onto ${\mathbb {C}}_-$ by reflection,

3.13 $$\begin{aligned} \log (N(z)):=\bigl (\log (N({\overline{z}}))\bigr )^*,\quad z\in {\mathbb {C}}_- \end{aligned}$$

(cf. (3.9)). By [26, Lemma 2.8] the function $z \mapsto \log (N(z))$ is also an ${\mathcal {L}}({\mathcal {G}})$-valued Nevanlinna function and satisfies

3.14 $$\begin{aligned} 0\le \mathrm {Im}(\log (N(z)))\le \pi I_{\mathcal {G}},\quad z\in {\mathbb {C}}_+. \end{aligned}$$

The following theorem is a variant and slight extension of [26, Theorem 2.10], the new and important feature here is that we provide a sufficient condition in terms of the function *N* such that $\log (N)$ admits an analytic continuation by reflection with respect to some real interval and a corresponding integral representation there.

### Theorem 3.3

Let $N:{\mathbb {C}}\backslash {\mathbb {R}}\rightarrow {\mathcal {L}}({\mathcal {G}})$ be a Nevanlinna function and assume that $N(z)^{-1}\in {\mathcal {L}}({\mathcal {G}})$ for some, and hence for all $z\in {\mathbb {C}}\backslash {\mathbb {R}}$. Then there exists a weakly Lebesgue measurable operator-valued function $\lambda \mapsto \Xi (\lambda )\in {\mathcal {L}}({\mathcal {G}})$ on ${\mathbb {R}}$ such that

3.15 $$\begin{aligned} \Xi (\lambda )=\Xi (\lambda )^*\, \text { and } \, 0\le \Xi (\lambda )\le I_{\mathcal {G}}\, \text { for a.e.}~\lambda \in {\mathbb {R}}, \end{aligned}$$

and the Nevanlinna function $\log (N):{\mathbb {C}}\backslash {\mathbb {R}}\rightarrow {\mathcal {L}}({\mathcal {G}})$ in (3.12)–(3.13) admits an integral representation of the form

3.16 $$\begin{aligned} \log (N(z))= C +\int _{\mathbb {R}}\left( \frac{1}{\lambda - z}-\frac{\lambda }{1+\lambda ^2}\right) \Xi (\lambda )\, d\lambda , \end{aligned}$$

where $C=\mathrm {Re}(\log (N(i)))\in {\mathcal {L}}({\mathcal {G}})$ is a self-adjoint operator and the integral is understood in the weak sense.

If, in addition, *N* admits an analytic continuation by reflection with respect to an open interval $I\subset {\mathbb {R}}$ such that $\sigma (N(z))\subset (\varepsilon ,\infty )$ for some $\varepsilon >0$ and all $z \in I$, then also $\log (N)$ admits an analytic continuation by reflection with respect to *I*, $\Xi (\lambda )=0$ for a.e. $\lambda \in I$, and (3.16) remains valid for $z \in I$.

### Proof

We make use of the representation (3.10) applied to the Nevanlinna function $\log (N)$ with $z_0=i$. Then there exists a Hilbert space ${\mathcal {K}}$ and $R\in {\mathcal {L}}({\mathcal {G}},{\mathcal {K}})$ such that

3.17 $$\begin{aligned} \log (N(z)) = C+ z R^* R + (1 + z^2)R^*(L - z I_{{\mathcal {K}}})^{-1}R, \quad z \in {\mathbb {C}}\backslash {\mathbb {R}}, \end{aligned}$$

where $C=\mathrm {Re}(\log (N(i))) \in {\mathcal {L}}({\mathcal {G}})$ is a self-adjoint operator. For $h\in {\mathcal {G}}$ it follows from (3.17) and (3.14) that

$$\begin{aligned} \lim _{y\rightarrow +\infty }\frac{1}{y}\mathrm {Re}\bigl (\log ((N(iy))h,h)_{{\mathcal {G}}})\bigr ) =0 = \lim _{y\rightarrow +\infty } \frac{1}{y}\mathrm {Im}\bigl (\log ((N(iy))h,h)_{{\mathcal {G}}})\bigr ), \end{aligned}$$

so that (3.11) holds for the function $\log (N)$. Hence *L* in (3.17) is a self-adjoint operator in ${\mathcal {K}}$ (cf. [26, Lemma 2.9]). We can assume that the model is chosen minimal and hence $\rho (L)$ coincides with the maximal domain of analyticity of the Nevanlinna function $\log (N)$.

In order to prove (3.15) and (3.16) one can argue in the same way as in the proof of [26, Theorem 2.10]. Let $\lambda \mapsto E_L(\lambda )$ be the spectral function of *L* such that $\lim _{\lambda \downarrow -\infty } (E_L(\lambda )h,h)_{{\mathcal {G}}}=0$. Then (3.17) yields

$$\begin{aligned} \bigl (\log (N(z))h,h\bigr )_{{\mathcal {G}}} = (Ch,h)_{{\mathcal {G}}}+\int _{\mathbb {R}}\left( \frac{1}{\lambda - z} - \frac{\lambda }{1 + \lambda ^2}\right) (1 + \lambda ^2)\, d \bigl (R^*E_L(\lambda )Rh,h\bigr )_{{\mathcal {G}}} \end{aligned}$$

for $h\in {\mathcal {G}}$, $z \in {\mathbb {C}}\backslash {\mathbb {R}}$, and (3.14) and the Stieltjes inversion formula imply that the measures

3.18 $$\begin{aligned} d \omega _h(\cdot )=(1 + \lambda ^2)d \bigl (R^*E_L(\cdot )Rh,h\bigr )_{{\mathcal {G}}} \end{aligned}$$

are absolutely continuous with respect to the Lebesgue measure $d\lambda$ and there exist measurable functions $\xi _h$ with $0\le \xi _h(\lambda )\le \Vert h\Vert ^2_{{\mathcal {G}}}$ for a.e. $\lambda \in {\mathbb {R}}$ such that $d\omega _h(\lambda )=\xi _h(\lambda )\, d\lambda$. Hence there exists a weakly Lebesgue measurable function $\lambda \mapsto \Xi (\lambda )$ such that

$$\begin{aligned} \xi _h(\lambda )=(\Xi (\lambda )h,h)_{{\mathcal {G}}}\, \text { and } \, 0\le \Xi (\lambda )\le I_{\mathcal {G}}, \end{aligned}$$

proving (3.15) and (3.16).

Next, assume that *N* admits an analytic continuation by reflection with respect to an open interval $I\subset {\mathbb {R}}$ such that $\sigma (N(z))\subset (\varepsilon ,\infty )$ for some $\varepsilon >0$ and all $z \in I$. Fix some $z_0 \in I$ and an open ball ${\mathcal {B}}_{z_0}\subset {\mathbb {C}}$ centered at $z_0$ such that *N* is analytic on ${\mathcal {B}}_{z_0}$. Since $\sigma (N(z_0))\subset (\varepsilon ,\infty )$ we can assume that ${\mathcal {B}}_{z_0}$ was chosen such that

$$\begin{aligned} \sigma (N(z))\subset \{z\in {\mathbb {C}}\, | \, \varepsilon /2< \mathrm {Re}(z), \, 0 \le \mathrm {Im}(z) < \varepsilon \}, \quad z \in {\mathcal {B}}_{z_0}\cap {\mathbb {C}}_+, \end{aligned}$$

and hence the operators *N*(*z*), $z \in {\mathcal {B}}_{z_0}\cap {\mathbb {C}}_+$, satisfy the assumptions in Lemma 3.1. Therefore, the operators

$$\begin{aligned} \log (N(z)^*):=-i\int _0^\infty \bigl [(N(z)^*+ i \lambda I_{{\mathcal {G}}})^{-1}-(1+ i \lambda )^{-1}I_{\mathcal {G}}\bigr ] \, d\lambda , \quad z \in {\mathcal {B}}_{z_0}\cap {\mathbb {C}}_+, \end{aligned}$$

are well-defined, and since $N({\overline{z}})=N(z)^*$, it follows that

$$\begin{aligned} \log (N(z))=-i\int _0^\infty \bigl [(N(z)+ i \lambda I_{{\mathcal {G}}})^{-1}-(1+ i \lambda )^{-1}I_{\mathcal {G}}\bigr ] \, d\lambda , \quad z \in {\mathcal {B}}_{z_0}\cap {\mathbb {C}}_-, \end{aligned}$$

are well-defined, bounded operators in ${\mathcal {G}}$. Furthermore, Lemma 3.1 also ensures that for $z \in {\mathcal {B}}_{z_0}\cap {\mathbb {R}}$ the operators

$$\begin{aligned} \log (N(z)):=-i\int _0^\infty \bigl [(N(z)+ i \lambda I_{{\mathcal {G}}})^{-1}-(1+ i \lambda )^{-1}I_{\mathcal {G}}\bigr ] \, d\lambda , \quad z \in {\mathcal {B}}_{z_0}\cap {\mathbb {R}}, \end{aligned}$$

are well-defined, bounded operators in ${\mathcal {G}}$. Thus for all $z \in {\mathcal {B}}_{z_0}$, the operators $\log (N(z))$ are well-defined via (3.12). It then follows from (3.12) that the function $z \mapsto \log (N(z))$ is analytic on ${\mathcal {B}}_{z_0}$ (cf. [26, Proof of Lemma 2.8]).

We shall now also make use of the logarithm

3.19 $$\begin{aligned} \ln (z)=\int _{-\infty }^0\left( \frac{1}{\lambda - z}-\frac{\lambda }{1 + \lambda ^2}\right) d\lambda , \quad z\in {\mathbb {C}}\backslash (-\infty ,0], \end{aligned}$$

which corresponds to the cut along the negative real axis. Since

$$\begin{aligned} \sigma (N(z))\subset \{ z\in {\mathbb {C}}\, | \, \varepsilon /2< \mathrm {Re}(z), \, -\varepsilon< \mathrm {Im}(z) < \varepsilon \},\quad z \in {\mathcal {B}}_{z_0}, \end{aligned}$$

it follows that

3.20 $$\begin{aligned} \ln (N(z))=\int _{-\infty }^0 \bigl [(\lambda I_{{\mathcal {G}}}-N(z))^{-1} - \lambda (1 + \lambda ^2)^{-1}I_{\mathcal {G}}\bigr ] \, d\lambda , \quad z \in {\mathcal {B}}_{z_0}, \end{aligned}$$

are well-defined operators and the function $z \mapsto \ln (N(z))$ is analytic on ${\mathcal {B}}_{z_0}$. In addition, (3.20) yields

3.21 $$\begin{aligned} \bigl (\ln (N(z))\bigr )^*=\ln (N(z)^*),\quad z \in {\mathcal {B}}_{z_0}. \end{aligned}$$

As $\log (z)=\ln (z)$ (see (3.1)) for all $z>0$ and *N*(*z*) is self-adjoint for $z \in I$ it follows from the spectral theorem that

$$\begin{aligned} \log (N(z))=\ln (N(z)),\quad z \in I, \end{aligned}$$

and hence $\log (N(z))=\ln (N(z))$, $z \in {\mathcal {B}}_{z_0}$, by analyticity. Therefore, (3.21) and $N(z)^*=N({\overline{z}})$ yield

$$\begin{aligned} \bigl (\log (N(z))\bigr )^*=\bigl (\ln (N(z))\bigr )^*=\ln (N(z)^*)=\ln (N({\overline{z}}))=\log (N({\overline{z}})), \quad z \in {\mathcal {B}}_{z_0}. \end{aligned}$$

It follows that $z \mapsto \log (N(z))$ is analytic on ${\mathcal {B}}_{z_0}$ and the continuation of $\log (N)$ onto ${\mathcal {B}}_{z_0} \cap {\mathbb {C}}_-$ coincides with the extension of $\log (N)$ onto ${\mathbb {C}}_-$ defined by

$$\begin{aligned} \log (N(z))=\bigl (\log N({\overline{z}})\bigr )^*,\quad z\in {\mathbb {C}}_-, \end{aligned}$$

(cf. (3.9)). This reasoning applies to all $\nu \in I$ and hence we have shown that $\log (N)$ admits an analytic continuation by reflection with respect to *I*.

Since the operator model for $\log (N)$ is minimal the interval *I* belongs to $\rho (L)$ and the representation (3.17) remains valid for $z \in \rho (L)$. It follows that the measures $d\omega _h(\cdot )$, $h\in {\mathcal {G}}$, in (3.18) have no support in *I* and hence their Radon–Nikodym deriatives satisfy $\xi _h(\lambda )=0$ for a.e. $\lambda \in I$. It follows that $(\Xi (\lambda )h,h)_{{\mathcal {G}}}=0$ for a.e. $\lambda \in I$ and all $h\in {\mathcal {G}}$. Since $\Xi (\lambda )\ge 0$ we conclude $\Xi (\lambda )=0$ for a.e. $\lambda \in I$. $\square$

In the next proposition we provide a sufficient condition such that the values of the function $\Xi$ are trace class operators and we express the traces of $\Xi (\lambda )$ in terms of certain weak limits of the imaginary part of $\log (N)$.

### Proposition 3.4

Let $N:{\mathbb {C}}\backslash {\mathbb {R}}\rightarrow {\mathcal {L}}({\mathcal {G}})$ be a Nevanlinna function such that $N(z)^{-1}\in {\mathcal {L}}({\mathcal {G}})$ for some, and hence for all $z\in {\mathbb {C}}\backslash {\mathbb {R}}$, and assume that *N* admits an analytic continuation by reflection with respect to an open interval $I\subset {\mathbb {R}}$ such that $\sigma (N(\zeta ))\subset (\varepsilon ,\infty )$ for some $\varepsilon >0$ and all $\zeta \in I$. Consider

3.22 $$\begin{aligned} \log (N(z))= C +\int _{\mathbb {R}}\left( \frac{1}{\lambda - z}-\frac{\lambda }{1 + \lambda ^2}\right) \Xi (\lambda )\, d\lambda , \end{aligned}$$

for $z\in ({\mathbb {C}}\backslash {\mathbb {R}})\cup I$ with $\Xi (\lambda )=\Xi (\lambda )^*$ and $0\le \Xi (\lambda )\le I_{\mathcal {G}}$ for a.e. $\lambda \in {\mathbb {R}}$ as in (3.15), and assume, in addition, that for some $k \in {\mathbb {N}}_0$ and some $\zeta \in I$,

3.23 $$\begin{aligned} \frac{d^{2k+1}}{d\zeta ^{2k+1}}\log (N(\zeta )) \in {\mathfrak S}_1({\mathcal {G}}). \end{aligned}$$

Then $0\le \Xi (\lambda )\in {\mathfrak S}_1({\mathcal {G}})$ for a.e. $\lambda \in {\mathbb {R}}$, and

3.24 $$\begin{aligned} {{\mathrm{tr}}}_{{\mathcal {G}}}(\Xi (\lambda ))=\sum _{j \in J} \lim _{\varepsilon \downarrow 0}\frac{1}{\pi }\bigl (\mathrm {Im}(\log (N(\lambda +i\varepsilon )))\varphi _j,\varphi _j\bigr )_{\mathcal {G}}\end{aligned}$$

holds for any orthonormal basis $(\varphi _j)_{j \in J}$ in ${\mathcal {G}}$ $(J \subseteq {\mathbb {N}}$ an appropriate index set ) and for a.e. $\lambda \in {\mathbb {R}}$. Furthermore, if (3.23) holds for some $\zeta \in I$ and $k=0$, that is,

3.25 $$\begin{aligned} \frac{d}{d\zeta }\log (N(\zeta )) \in {\mathfrak S}_1({\mathcal {G}}), \end{aligned}$$

then $\mathrm {Im}(\log (N(z)))\in {\mathfrak S}_1({\mathcal {G}})$ for all $z\in {\mathbb {C}}\backslash {\mathbb {R}}$, the limit

$$\begin{aligned} \mathrm {Im}\bigl (\log (N(\lambda +i0))\bigr ):=\lim _{\varepsilon \downarrow 0}\mathrm {Im}\big (\log (N(\lambda +i\varepsilon ))\big )\in {\mathfrak S}_1({\mathcal {G}}) \end{aligned}$$

exists for a.e. $\lambda \in {\mathbb {R}}$ in the norm of ${\mathfrak S}_1({\mathcal {G}})$, and

3.26 $$\begin{aligned} {{\mathrm{tr}}}_{{\mathcal {G}}}(\Xi (\lambda ))=\frac{1}{\pi }{{\mathrm{tr}}}_{\mathcal {G}}\bigl (\mathrm {Im}(\log (N(\lambda +i0)))\bigr ) \quad \text { for a.e.}~\lambda \in {\mathbb {R}}. \end{aligned}$$

### Proof

The assumption (3.23) together with the integral representation (3.22) yields

3.27 $$\begin{aligned}&\frac{d^{2k+1}}{d\zeta ^{2k+1}}\log (N(\zeta ))\nonumber \\&\quad = (2k+1)!\int _{\mathbb {R}}\frac{1}{(\lambda - \zeta )^{2k+2}}\,\Xi (\lambda )\, d\lambda \in {\mathfrak S}_1({\mathcal {G}}), \quad k \in {\mathbb {N}}_0, \; \zeta \in I. \end{aligned}$$

Since $\Xi (\lambda )\ge 0$ by (3.15) and $(\lambda - \zeta )^{-2k-2}\ge 0$ for all $\lambda \in {\mathbb {R}}$, $\zeta \in I$, it follows together with the assumption (3.23) that the integral in (3.27) is a nonnegative trace class operator. Similarly, as in [26, Proof of Theorem 2.10], the monotone convergence theorem yields $\Xi (\lambda )\in {\mathfrak S}_1({\mathcal {G}})$ for a.e. $\lambda \in {\mathbb {R}}$. For $\varepsilon >0$ it follows from the integral representation (3.22) that

3.28 $$\begin{aligned} \bigl (\mathrm {Im}(\log (N(\lambda +i\varepsilon )))h,h\bigr )_{\mathcal {G}}= \int _{\mathbb {R}}\frac{\varepsilon }{\vert \lambda ' - \lambda \vert ^2+\varepsilon ^2}\,(\Xi (\lambda ')h,h)_{\mathcal {G}}\,d\lambda ' \end{aligned}$$

holds for all $h\in {\mathcal {G}}$ and all $\lambda \in {\mathbb {R}}$, and therefore the Stietljes inversion formula yields

3.29 $$\begin{aligned} \lim _{\varepsilon \downarrow 0}\frac{1}{\pi }\bigl (\mathrm {Im}(\log (N(\lambda +i\varepsilon )))h,h\bigr )_{\mathcal {G}}= (\Xi (\lambda )h,h)_{\mathcal {G}}\quad \text { for a.e.}~\lambda \in {\mathbb {R}}. \end{aligned}$$

Let $(\varphi _j)_{j \in J}$ be an orthonormal basis in ${\mathcal {G}}$. Then

3.30 $$\begin{aligned} \lim _{\varepsilon \downarrow 0}\frac{1}{\pi }\bigl (\mathrm {Im}(\log (N(\lambda +i\varepsilon )))\varphi _j,\varphi _j\bigr )_{\mathcal {G}}= (\Xi (\lambda )\varphi _j,\varphi _j)_{\mathcal {G}}\end{aligned}$$

holds for all $\lambda \in {\mathbb {R}}\backslash {\mathcal {A}}_j$, where ${\mathcal {A}}_j\subset {\mathbb {R}}$, $j \in J$, is a set of Lebesgue measure zero. The countable union ${\mathcal {A}}:=\cup _{j \in J}{\mathcal {A}}_j$ is also a set of Lebesgue measure zero and for all $\lambda \in {\mathbb {R}}\backslash {\mathcal {A}}$ and all $\varphi _j$ one has (3.30). Taking into acount that $0\le \Xi (\lambda )\in {\mathfrak S}_1({\mathcal {G}})$ for a.e. $\lambda \in {\mathbb {R}}$ this implies

$$\begin{aligned} \sum _{j \in J} \lim _{\varepsilon \downarrow 0}\frac{1}{\pi }\bigl (\mathrm {Im}(\log (N(\lambda +i\varepsilon )))\varphi _j,\varphi _j\bigr )_{\mathcal {G}}= \sum _{j \in J} (\Xi (\lambda )\varphi _j,\varphi _j)_{\mathcal {G}}={{\mathrm{tr}}}_{{\mathcal {G}}}(\Xi (\lambda )) \end{aligned}$$

for a.e. $\lambda \in {\mathbb {R}}$, that is, (3.24) holds.

In the special case that (3.23) holds with $k=0$ the formula (3.27) has the form

$$\begin{aligned} \frac{d}{d\zeta }\log (N(\zeta )) = \int _{\mathbb {R}}\frac{1}{(\lambda - \zeta )^{2}}\,\Xi (\lambda )\, d\lambda \in {\mathfrak S}_1({\mathcal {G}}), \quad \zeta \in I. \end{aligned}$$

Since $0\le \Xi (\lambda )\in {\mathfrak S}_1({\mathcal {G}})$ for a.e. $\lambda \in {\mathbb {R}}$ we conclude

3.31 $$\begin{aligned} \mathrm {Im}(\log (N(z)))= \int _{\mathbb {R}}\frac{\mathrm {Im}(z)}{\vert \lambda - z\vert ^2}\,\Xi (\lambda )\,d\lambda \in {\mathfrak S}_1({\mathcal {G}}) \end{aligned}$$

for all $z\in {\mathbb {C}}\backslash {\mathbb {R}}$. The last assertion on the existence of the limit $\mathrm {Im}(\log (N(\lambda +i0)))$ for a.e. $\lambda \in {\mathbb {R}}$ in ${\mathfrak S}_1({\mathcal {G}})$ is an immediate consequence of (3.31) and well-known results in [10, 60, 61] (cf. [26, Theorem 2.2(iii)]). $\square$

The following lemma will be useful in the proof of our main result, Theorem 4.1, in the next section; it also provides a sufficient condition for the assumption (3.23) in Proposition 3.4.

### Lemma 3.5

Let $N:{\mathbb {C}}\backslash {\mathbb {R}}\rightarrow {\mathcal {L}}({\mathcal {G}})$ be a Nevanlinna function such that $N(z)^{-1}\in {\mathcal {L}}({\mathcal {G}})$ for some, and hence for all $z\in {\mathbb {C}}\backslash {\mathbb {R}}$. Let $\ell \in {\mathbb {N}}$ and assume that

3.32 $$\begin{aligned} \frac{d^j}{dz^j} N (z)\in {\mathfrak S}_\frac{l}{j}({\mathcal {G}}),\quad j=1,\dots ,\ell , \end{aligned}$$

holds for all $z\in {\mathbb {C}}\backslash {\mathbb {R}}$. Then

3.33 $$\begin{aligned} \frac{d^{\ell }}{dz^{\ell }}\log (N(z))\in {\mathfrak S}_1({\mathcal {G}})\quad \text { and } \quad \frac{d^{\ell -1}}{dz^{\ell -1}}\left( N(z)^{-1}\frac{d}{dz} N (z)\right) \in {\mathfrak S}_1({\mathcal {G}}) \end{aligned}$$

and

3.34 $$\begin{aligned} {{\mathrm{tr}}}_{{\mathcal {G}}}\left( \frac{d^{\ell -1}}{dz^{\ell -1}}\left( N(z)^{-1}\frac{d}{dz} N(z)\right) \right) ={{\mathrm{tr}}}_{{\mathcal {G}}}\left( \frac{d^{\ell }}{dz^{\ell }}\log (N(z))\right) \end{aligned}$$

hold for all $z\in {\mathbb {C}}\backslash {\mathbb {R}}$.

Furthermore, if *N* admits an analytic continuation by reflection with respect to an open interval $I\subset {\mathbb {R}}$ such that $\sigma (N(z))\subset (\varepsilon ,\infty )$ for some $\varepsilon >0$ and all $z \in I$, and (3.32) is satisfied for $z \in I$, then also the assertions (3.33) and (3.34) are valid for all $z \in I$.

### Proof

We prove Lemma 3.5 for the case $\ell =1$ and leave the induction step to the reader. Assume that

3.35 $$\begin{aligned} \frac{d}{dz} N (z)\in {\mathfrak S}_1({\mathcal {G}}) \end{aligned}$$

holds for $z\in {\mathbb {C}}_+$ (the proof works also for $z \in I$ if *N* admits an analytic continuation by reflection with respect to *I* and $\sigma (N(z))\subset (\varepsilon ,\infty )$ holds for some $\varepsilon >0$ and all $z \in I$). One notes that $N(z)^{-1}\in {\mathcal {L}}({\mathcal {G}})$ implies the second assertion in (3.33) for $\ell =1$. In addition, one observes that $\log (N(z))$ is well-defined and analytic for $z\in {\mathbb {C}}_+$ according to (3.12) and Theorem 3.3. Since $0\in \rho (N(z))$ and

3.36 $$\begin{aligned} \big \Vert (N(z)+ i \lambda I_{{\mathcal {G}}})^{-1}\big \Vert _{{\mathcal {L}}({\mathcal {G}})} \le \lambda ^{-1},\quad \lambda > 0 \end{aligned}$$

(cf. the proof of Lemma 3.1 and [26, Proof of Lemma 2.6 (*i*)]), it follows by the dominated convergence theorem that

$$\begin{aligned}&\frac{d}{dz} \bigl (\log (N(z))\varphi ,\psi \bigr )_{{\mathcal {G}}}\\&\quad =i\int _0^\infty \left( (N(z)+ i \lambda I_{{\mathcal {G}}})^{-1} \left( \frac{d}{dz} N(z)\right) (N(z)+ i \lambda I_{{\mathcal {G}}})^{-1}\varphi ,\psi \right) _{{\mathcal {G}}} d\lambda \end{aligned}$$

holds for all $\varphi ,\psi \in {\mathcal {G}}$ and all $z\in {\mathbb {C}}_+$, and hence

3.37 $$\begin{aligned}&\frac{d}{dz}\log (N(z))\nonumber \\&\quad =i\int _0^\infty (N(z)+ i \lambda I_{{\mathcal {G}}})^{-1} \left( \frac{d}{dz} N(z)\right) (N(z)+ i \lambda I_{{\mathcal {G}}})^{-1}\, d\lambda , \quad z\in {\mathbb {C}}_+. \end{aligned}$$

The assumption (3.35) yields

$$\begin{aligned} (N(z)+ i \lambda I_{{\mathcal {G}}})^{-1} \left( \frac{d}{dz} N(z)\right) (N(z)+ i \lambda I_{{\mathcal {G}}})^{-1}\in {\mathfrak S}_1({\mathcal {G}}), \quad \lambda \ge 0. \end{aligned}$$

From (3.36) and the properties of the trace class norm $\Vert \cdot \Vert _{{\mathfrak S}_1({\mathcal {G}})}$ one gets

$$\begin{aligned}&\left\| (N(z)+ i \lambda I_{{\mathcal {G}}})^{-1} \left( \frac{d}{dz} N(z)\right) (N(z)+ i \lambda I_{{\mathcal {G}}})^{-1}\right\| _{{\mathfrak S}_1({\mathcal {G}})}\le \frac{1}{\lambda ^2}\left\| \frac{d}{dz} N(z)\right\| _{{\mathfrak S}_1({\mathcal {G}})}, \end{aligned}$$

$\lambda > 0,$ and hence the integral in (3.37) exists in trace class norm, that is, the first assertion in (3.33) holds for $\ell =1$. In order to prove (3.34) for $\ell =1$ we use (3.37) and cyclicity of the trace (i.e., ${{\mathrm{tr}}}_{{\mathcal {G}}}(CD) = {{\mathrm{tr}}}_{{\mathcal {G}}}(DC)$ whenever $C,D\in {\mathcal {L}}({\mathcal {G}})$ such that $CD,DC\in {\mathfrak S}_1({\mathcal {G}})$) and obtain

$$\begin{aligned} \begin{aligned} {{\mathrm{tr}}}_{{\mathcal {G}}}\left( \frac{d}{dz}\log (N(z))\right)&={{\mathrm{tr}}}_{{\mathcal {G}}}\left( i\int _0^\infty (N(z)+ i \lambda I_{{\mathcal {G}}})^{-1} \left( \frac{d}{dz} N(z)\right) (N(z)+ i \lambda I_{{\mathcal {G}}})^{-1}\, d\lambda \right) \\&=i\int _0^\infty {{\mathrm{tr}}}_{{\mathcal {G}}}\left( (N(z)+ i \lambda I_{{\mathcal {G}}})^{-2} \frac{d}{dz} N(z)\right) \, d\lambda \\&=\int _0^\infty {{\mathrm{tr}}}_{{\mathcal {G}}}\left( -\frac{d}{d\lambda }(N(z)+ i \lambda I_{{\mathcal {G}}})^{-1} \frac{d}{dz} N(z)\right) \, d\lambda \\&=-\int _0^\infty \frac{d}{d\lambda }{{\mathrm{tr}}}_{{\mathcal {G}}}\left( (N(z)+ i \lambda I_{{\mathcal {G}}})^{-1} \frac{d}{dz} N(z)\right) \, d\lambda \\&={{\mathrm{tr}}}_{{\mathcal {G}}}\left( N(z)^{-1} \frac{d}{dz} N(z)\right) . \end{aligned} \end{aligned}$$

Here we have used $\lim _{\lambda \rightarrow +\infty } {{\mathrm{tr}}}_{{\mathcal {G}}}\big ((N(z)+ i \lambda I_{{\mathcal {G}}})^{-1} \frac{d}{dz} N(z)\big )= 0$ in the last step, which follows from

$$\begin{aligned} \left\| (N(z)+ i \lambda I_{{\mathcal {G}}})^{-1} \frac{d}{dz} N(z)\right\| _{{\mathfrak S}_1({\mathcal {G}})} \le \frac{1}{\lambda }\left\| \frac{d}{dz} N(z)\right\| _{{\mathfrak S}_1({\mathcal {G}})}, \quad \lambda > 0. \end{aligned}$$

$$\square $$

## A representation of the spectral shift function in terms of the Weyl function

Let *A* and *B* be self-adjoint operators in a separable Hilbert space ${\mathfrak H}$ and assume that the closed symmetric operator $S=A\cap B$, that is,

4.1 $$\begin{aligned} Sf=Af=Bf,\quad {{\mathrm{dom}}}(S) = \bigl \{f\in {{\mathrm{dom}}}(A)\cap {{\mathrm{dom}}}(B) \, | \, Af=Bf\bigr \}, \end{aligned}$$

is densely defined. According to Proposition 2.4 we can choose a quasi boundary triple $\{{\mathcal {G}},\Gamma _0,\Gamma _1\}$ with $\gamma$-field $\gamma$ and Weyl function *M* such that

4.2 $$\begin{aligned} A=T\upharpoonright \ker (\Gamma _0)\quad \text { and } \quad B=T\upharpoonright \ker (\Gamma _1), \end{aligned}$$

and

4.3 $$\begin{aligned} (B - z I_{{\mathfrak H}})^{-1}-(A - z I_{{\mathfrak H}})^{-1}=-\gamma (z) M(z)^{-1}\gamma ({\overline{z}})^*,\quad z \in \rho (A)\cap \rho (B). \end{aligned}$$

In the next theorem we find an explicit expression for a spectral shift function of the pair $\{A,B\}$ in terms of the Weyl function *M*, see [49, Theorem 1] for the case that the difference of (the first powers of) the resolvents *A* and *B* is a rank one operator, [7, Theorem 4.1] for the finite-rank case, and [56, Theorem 3.4 and Remark 3.5] for a different representation via a perturbation determinant involving the Weyl function and boundary parameters of an ordinary boundary triple. In the present situation of infinite dimensional perturbations and differences of higher powers of resolvents a much more careful analysis is necessary, in particular, the properties of the logarithm of operator-valued Nevanlinna functions discussed in Sect. 3 will play an essential role. In Theorem 4.1 an implicit sign condition on the perturbation is imposed via the resolvents which leads to a nonnegative spectral shift function; this condition will be weakend afterwards (cf. (4.25) and (4.29)). In the special case that *A* and *B* are semibounded operators the sign condition (4.4) is equivalent to the inequality $\mathfrak t_A\le \mathfrak t_B$ of the semibounded closed quadratic forms $\mathfrak t_A$ and $\mathfrak t_B$ corresponding to *A* and *B*. In order to ensure that for some $k \in {\mathbb {N}}_0$ the difference of the $2k+1$th-powers of the resolvents of *A* and *B* is a trace class operator a set of ${\mathfrak S}_p$-conditions on the $\gamma$-field and the Weyl function are imposed. In the applications in Sects. 5 and 6 these conditions are satisfied.

### Theorem 4.1

Let *A* and *B* be self-adjoint operators in a separable Hilbert space ${\mathfrak H}$ and assume that for some $\zeta _0 \in \rho (A)\cap \rho (B)\cap {\mathbb {R}}$ the sign condition

4.4 $$\begin{aligned} (A-\zeta _0 I_{{\mathfrak H}})^{-1}\ge (B- \zeta _0 I_{{\mathfrak H}})^{-1} \end{aligned}$$

holds. Let the closed symmetric operator $S=A\cap B$ in (4.1) be densely defined and let $\{{\mathcal {G}},\Gamma _0,\Gamma _1\}$ be a quasi boundary triple with $\gamma$-field $\gamma$ and Weyl function *M* such that (4.2), and hence also (4.3), hold. Assume that $M(z_1)$, $M(z_2)^{-1}$ are bounded (not necessarily everywhere defined ) operators in ${\mathcal {G}}$ for some $z_1, z_2 \in \rho (A)\cap \rho (B)$ and that for some $k \in {\mathbb {N}}_0$, all $p,q \in {\mathbb {N}}_0$, and all $z \in \rho (A)\cap \rho (B)$,

4.5 $$\begin{aligned} \left( \frac{d^p}{d z^p}\overline{\gamma (z)}\right) \frac{d^q}{dz^q}\bigl ( M(z)^{-1} \gamma ({\overline{z}})^*\bigr )\in {\mathfrak S}_1({\mathfrak H}),\quad p+q=2k, \end{aligned}$$

4.6 $$\begin{aligned} \left( \frac{d^q}{dz^q}\bigl ( M(z)^{-1} \gamma ({\overline{z}})^*\bigr )\right) \frac{d^p}{dz^p}\overline{\gamma (z)}\in {\mathfrak S}_1({\mathcal {G}}),\quad p+q=2k, \end{aligned}$$

and

4.7 $$\begin{aligned} \frac{d^j}{dz^j} \overline{M (z)}\in {\mathfrak S}_{(2k+1)/j}({\mathcal {G}}),\quad j=1,\dots ,2k+1. \end{aligned}$$

Then the following assertions (i) and (ii) hold:

(i) The difference of the $2k+1$th-powers of the resolvents of *A* and *B* is a trace class operator, that is,
  4.8 $$\begin{aligned} \big [(B - z I_{{\mathfrak H}})^{-(2k+1)}-(A - z I_{{\mathfrak H}})^{-(2k+1)}\big ] \in {\mathfrak S}_1({\mathfrak H}) \end{aligned}$$
  holds for all $z\in \rho (A)\cap \rho (B)$.
(ii) For any orthonormal basis $(\varphi _j)_{j \in J}$ in ${\mathcal {G}}$ the function
  4.9 $$\begin{aligned} \xi (\lambda )= \sum _{j \in J} \lim _{\varepsilon \downarrow 0}\pi ^{-1}\bigl (\mathrm {Im}\big (\log \big (\overline{M(\lambda +i\varepsilon )}\big )\big )\varphi _j,\varphi _j\bigr )_{\mathcal {G}}\quad \text { for a.e.}~\lambda \in {\mathbb {R}}, \end{aligned}$$
  is a spectral shift function for the pair $\{A,B\}$ such that $\xi (\lambda )=0$ in an open neighborhood of $\zeta _0$; the function $\xi$ does not depend on the choice of the orthonormal basis $(\varphi _j)_{j \in J}$. In particular, the trace formula
  $$\begin{aligned} {{\mathrm{tr}}}_{{\mathfrak H}}\bigl ( (B - z I_{{\mathfrak H}})^{-(2k+1)}-(A - z I_{{\mathfrak H}})^{-(2k+1)}\bigr ) = - (2k+1) \int _{\mathbb {R}}\frac{\xi (\lambda )\, d\lambda }{(\lambda - z)^{2k+2}} \end{aligned}$$
  is valid for all $z \in \rho (A)\cap \rho (B)$.

### Proof

*Step 1* In this step we show that the Nevanlinna function $z \mapsto \overline{M(z)}$ satisfies the assumptions of Theorem 3.3 and admits an analytic continuation by reflection with respect to an open interval $I_{\zeta _0}\subset {\mathbb {R}}$, such that $\sigma \big (\overline{M(z)}\big )\subset (\varepsilon ,\infty )$ for some $\varepsilon >0$ and all $z \in I_{\zeta _0}$, where $I_{\zeta _0}\subset \rho (A)\cap \rho (B)$ is a suitable small open interval in ${\mathbb {R}}$ with $\zeta _0 \in I_{\zeta _0}$. Hence by Theorem 3.3 there exists a weakly Lebesgue measurable operator function $\lambda \mapsto \Xi (\lambda )\in {\mathcal {L}}({\mathcal {G}})$ on ${\mathbb {R}}$ such that

4.10 $$\begin{aligned} \Xi (\lambda )=\Xi (\lambda )^*\quad \text { and } \quad 0\le \Xi (\lambda )\le I_{\mathcal {G}}\quad \text { for a.e.}~\lambda \in {\mathbb {R}}, \end{aligned}$$

and the Nevanlinna function $\log \big (\overline{M}\big )$ admits an integral representation of the form

4.11 $$\begin{aligned} \log \big (\overline{M(z)}\big ) = \mathrm {Re}\big (\log (\overline{M(i)}\big )\big ) +\int _{\mathbb {R}}\left( \frac{1}{\lambda - z}-\frac{\lambda }{1 + \lambda ^2}\right) \Xi (\lambda )\, d\lambda , \end{aligned}$$

valid for all $z\in ({\mathbb {C}}\backslash {\mathbb {R}})\cup I_{\zeta _0}$, and $\Xi (\lambda )=0$ for a.e. $\lambda \in I_{\zeta _0}$.

First, it follows from (2.8) and the assumption that $M(z_1)$ is bounded for some $z_1\in \rho (A)$ that *M*(*z*) is bounded for all $z \in \rho (A)$ and hence the closures are bounded operators defined on ${\mathcal {G}}$, that is,

4.12 $$\begin{aligned} \overline{M(z)} \in {\mathcal {L}}({\mathcal {G}}), \quad z \in \rho (A),\quad \text {and}\quad \mathrm {Im}\big (\overline{M(z)}\big ) \ge 0,\quad z\in {\mathbb {C}}_+, \end{aligned}$$

by (2.11). Since $-M^{-1}$ is the Weyl function corresponding to the quasi boundary triple $\{{\mathcal {G}},\Gamma _1,-\Gamma _0\}$, where $B=T\upharpoonright \ker (\Gamma _1)$ is self-adjoint according to (4.2), it follows from the assumption that $M(z_2)^{-1}$ is bounded for some $z_2\in \rho (B)$ that $M(z)^{-1}$ is bounded for all $z \in \rho (A)\cap \rho (B)$, that is,

4.13 $$\begin{aligned} \overline{M(z)^{-1}}\in {\mathcal {L}}({\mathcal {G}})\quad \text { for all } \, z \in \rho (A)\cap \rho (B). \end{aligned}$$

Therefore, taking into account (4.12) and (4.13), it follows that the logarithm $z \mapsto \log (\overline{M(z)})\in {\mathcal {L}}({\mathcal {G}})$ is well-defined by

4.14 $$\begin{aligned} \log \big (\overline{M(z)}\big ):=-i\int _0^\infty \bigl [\big (\overline{M(z)} + i \lambda I_{{\mathcal {G}}}\big )^{-1}-(1+ i \lambda )^{-1}I_{\mathcal {G}}\bigr ] \, d\lambda ,\quad z\in {\mathbb {C}}_+,\quad \end{aligned}$$

and

4.15 $$\begin{aligned} \log \big (\overline{M(z)}\big ):=\bigl (\log \big (\overline{M({\overline{z}})}\big )\bigr )^*,\quad z\in {\mathbb {C}}_-; \end{aligned}$$

see (3.12)–(3.13) in Sect. 3 and [26, Lemma 2.6]. We claim that the function $\overline{M}$ has the property

4.16 $$\begin{aligned} \sigma \big (\overline{M(z)}\big ) \subset (\varepsilon ,\infty ) \end{aligned}$$

for some $\varepsilon >0$ and all $z \in I_{\zeta _0}$, where $I_{\zeta _0}$ is a suitable small open interval in ${\mathbb {R}}$ with $\zeta _0 \in I_{\zeta _0}$. In fact, due to (4.3) and the sign condition (4.4), one has

$$\begin{aligned} 0&\le \bigl ((A - \zeta _0 I_{{\mathfrak H}})^{-1}f-(B - \zeta _0 I_{{\mathfrak H}})^{-1}f,f\bigr )_{{\mathfrak H}}\\&=\bigl (M(\zeta _0)^{-1}\gamma (\zeta _0)^*f,\gamma (\zeta _0)^*f\bigr )_{{\mathcal {G}}},\quad f\in {\mathfrak H}, \end{aligned}$$

and since ${{\mathrm{ran}}}(\gamma (\zeta _0)^*)$ is dense in ${\mathcal {G}}$ (see (2.3)), it follows that the bounded operator $M(\zeta _0)^{-1}$ is nonnegative. The same is true for $M(\zeta _0)$ and the closure $\overline{M(\zeta _0)}$, and from (4.13) one concludes $\sigma \big (\overline{M(\zeta _0)}\big )\subset (\varepsilon ,\infty )$ for some $\varepsilon >0$. Since $\zeta _0 \in \rho (A)\cap \rho (B)$ the Nevanlinna function $\overline{M}$ admits an analytic continuation by reflection with respect to a real neighborhood of $\zeta _0$, and it follows that (4.16) holds for all $\lambda$ in a sufficiently small interval $I_{\zeta _0} \subset \rho (A)\cap \rho (B)\cap {\mathbb {R}}$ with $\zeta _0 \in I_{\zeta _0}$.

*Step 2*  In this step we show that for $z\in ({\mathbb {C}}\backslash {\mathbb {R}})\cup I_{\zeta _0}$, the trace class property (4.8) holds, and that

4.17 $$\begin{aligned} {{\mathrm{tr}}}_{{\mathfrak H}}\bigl ((B - z I_{{\mathfrak H}})^{-(2k+1)}-(A - z I_{{\mathfrak H}})^{-(2k+1)}\bigr ) = {{\mathrm{tr}}}_{{\mathcal {G}}}\left( \frac{-1}{(2k)!}\frac{d^{2k+1}}{dz^{2k+1}} \log \big (\overline{M(z)}\big ) \right) . \end{aligned}$$

In fact, for $z\in ({\mathbb {C}}\backslash {\mathbb {R}})\cup I_{\zeta _0}$ one computes

$$\begin{aligned}&(B - z I_{{\mathfrak H}})^{-(2k+1)}-(A - z I_{{\mathfrak H}})^{-(2k+1)}\\&\quad =\frac{1}{(2k)!}\frac{d^{2k}}{dz^{2k}}\bigl ((B - z I_{{\mathfrak H}})^{-1} - (A - z I_{{\mathfrak H}})^{-1}\bigr )\\&\quad =\frac{-1}{(2k)!}\frac{d^{2k}}{dz^{2k}} \bigl (\gamma (z) M(z)^{-1}\gamma ({\overline{z}})^*\bigr ) =\frac{-1}{(2k)!}\frac{d^{2k}}{dz^{2k}} \bigl (\overline{\gamma (z)} M(z)^{-1}\gamma ({\overline{z}})^*\bigr )\\&\quad =\frac{-1}{(2k)!}\sum _{\begin{array}{c} p+q=2k \\ p,q\geqslant 0 \end{array}} \begin{pmatrix} 2k \\ p \end{pmatrix}\left( \frac{d^p}{d z^p}\,\overline{\gamma (z)}\right) \frac{d^q}{d z^q}\bigl ( M(z)^{-1} \gamma ({\overline{z}})^*\bigr ), \end{aligned}$$

and by assumption (4.5) each summand is a trace class operator; in the last step the product rule for holomorphic operator functions was applied, see, e.g. [6, (2.6)]. This proves (4.8). Furthermore, making use of both assumptions (4.5) and (4.6), the cyclicity of the trace (see, e.g., [72, Theorem 7.11(b)]), and

4.18 $$\begin{aligned} \frac{d}{dz} \overline{M(z)}=\gamma ({\overline{z}})^*\overline{\gamma (z)}, \quad z \in \rho (A), \end{aligned}$$

one obtains

$$\begin{aligned} \begin{aligned}&{{\mathrm{tr}}}_{{\mathfrak H}}\bigl ((B - z I_{{\mathfrak H}})^{-(2k+1)}-(A - z I_{{\mathfrak H}})^{-(2k+1)}\bigr )\\&\quad =\frac{-1}{(2k)!}\sum _{\begin{array}{c} p+q=2k \\ p,q\geqslant 0 \end{array}} \begin{pmatrix} 2k \\ p \end{pmatrix}{{\mathrm{tr}}}_{{\mathfrak H}}\left( \left( \frac{d^p}{d z^p}\,\overline{\gamma (z)}\right) \frac{d^q}{d z^q}\bigl ( M(z)^{-1} \gamma ({\overline{z}})^*\bigr )\right) \\&\quad =\frac{-1}{(2k)!}\sum _{\begin{array}{c} p+q=2k \\ p,q\geqslant 0 \end{array}} \begin{pmatrix} 2k \\ p \end{pmatrix}{{\mathrm{tr}}}_{{\mathcal {G}}}\left( \left( \frac{d^q}{d z^q}\bigl ( M(z)^{-1} \gamma ({\overline{z}})^*\bigr )\right) \frac{d^p}{d z^p}\,\overline{\gamma (z)}\right) \\&\quad ={{\mathrm{tr}}}_{{\mathcal {G}}}\left( \frac{-1}{(2k)!}\frac{d^{2k}}{dz^{2k}} \bigl (M(z)^{-1}\gamma ({\overline{z}})^*\overline{\gamma (z)}\bigr )\right) \\&\quad ={{\mathrm{tr}}}_{{\mathcal {G}}}\left( \frac{-1}{(2k)!}\frac{d^{2k}}{dz^{2k}} \left( \overline{M(z)^{-1}}\frac{d}{dz}\overline{M(z)}\right) \right) . \end{aligned} \end{aligned}$$

Noting that assumption (4.7) and Lemma 3.5 with $\ell =2k+1$ imply

4.19 $$\begin{aligned} \frac{d^{2k+1}}{dz^{2k+1}} \log \big (\overline{M(z)}\big )\in {\mathfrak S}_1({\mathcal {G}}), \end{aligned}$$

and that

4.20 $$\begin{aligned} {{\mathrm{tr}}}_{{\mathcal {G}}}\left( \frac{d^{2k}}{dz^{2k}}\left( \overline{M(z)^{-1}}\frac{d}{dz} \overline{M(z)}\right) \right) ={{\mathrm{tr}}}_{{\mathcal {G}}}\left( \frac{d^{2k+1}}{dz^{2k+1}}\log \big (\overline{M(z)}\big )\right) , \end{aligned}$$

one concludes the trace formula (4.17).

*Step 3*  Now we complete the proof of Theorem 4.1. Since (4.19) is valid for all $z \in I_{\zeta _0}$ the assumption (3.23) in Proposition 3.4 is satisfied. It then follows from Proposition 3.4 that $0 \le \Xi (\lambda )\in {\mathfrak S}_1({\mathcal {G}})$ for a.e. $\lambda \in {\mathbb {R}}$ and

4.21 $$\begin{aligned} {{\mathrm{tr}}}_{{\mathcal {G}}}(\Xi (\lambda ))=\sum _{j \in J} \lim _{\varepsilon \downarrow 0}\pi ^{-1}\bigl (\mathrm {Im}\big (\log \big (\overline{M(\lambda +i\varepsilon )}\big )\big )\varphi _j,\varphi _j\bigr )_{\mathcal {G}}\end{aligned}$$

holds for any orthonormal basis $(\varphi _j)_{j \in J}$ in ${\mathcal {G}}$ and for a.e. $\lambda \in {\mathbb {R}}$. Furthermore, from (4.11) one obtains

$$\begin{aligned} \frac{d^{2k+1}}{dz^{2k+1}}\log \big (\overline{M(z)}\big ) = (2k+1)! \int _{\mathbb {R}}\frac{1}{(\lambda - z)^{2k+2}}\, \Xi (\lambda )\, d\lambda , \quad z\in ({\mathbb {C}}\backslash {\mathbb {R}})\cup I_{\zeta _0}, \end{aligned}$$

and hence

4.22 $$\begin{aligned} {{\mathrm{tr}}}_{{\mathfrak H}}\bigl ((B - z I_{{\mathfrak H}})^{-(2k+1)}-(A - z I_{{\mathfrak H}})^{-(2k+1)}\bigr ) = - (2k+1) \int _{\mathbb {R}}\frac{{{\mathrm{tr}}}_{{\mathcal {G}}}(\Xi (\lambda ))\, d\lambda }{(\lambda - z)^{2k+2}} \end{aligned}$$

for all $z\in ({\mathbb {C}}\backslash {\mathbb {R}})\cup I_{\zeta _0}$ by (4.17). It also follows from (4.22) that

4.23 $$\begin{aligned} \int _{\mathbb {R}}\frac{{{\mathrm{tr}}}_{{\mathcal {G}}}(\Xi (\lambda )) \, d\lambda }{(1+\vert \lambda \vert )^{2k+2}} < \infty \end{aligned}$$

holds and together with (4.21)–(4.23) we conclude that the function

$$\begin{aligned} \xi (\lambda ):={{\mathrm{tr}}}_{{\mathcal {G}}}(\Xi (\lambda ))=\sum _{j \in J} \lim _{\varepsilon \downarrow 0}\pi ^{-1}\bigl (\mathrm {Im}\big (\log \big (\overline{M(\lambda +i\varepsilon )}\big )\big )\varphi _j,\varphi _j\bigr )_{\mathcal {G}}\quad \text { for a.e.}~\lambda \in {\mathbb {R}}\end{aligned}$$

in (4.9) is a spectral shift function for the pair $\{A,B\}$. Next, since

$${{\mathrm{tr}}}_{{\mathcal {G}}}(\Xi (\lambda ))= \sum _{j \in J}(\Xi (\lambda )\varphi _j,\varphi _j)_{\mathcal {G}}$$

does not depend on the choice of the orthonormal basis $(\varphi _j)_{j \in J}$, it follows that the function $\xi$ does not depend on the choice of the orthonormal basis (cf. Proposition 3.4). Finally, since $\Xi (\lambda )=0$ for a.e. $\lambda \in I_{\zeta _0}$ by Theorem 3.3 it follows that $\xi (\lambda )=0$ for a.e. $\lambda \in I_{\zeta _0}$. $\square$

In the special case $k=0$ Theorem 4.1 can be slightly improved. Here the essential feature is that Proposition 3.4 can be applied under the assumption (3.25), so that the limit $\mathrm {Im}(\log (\overline{M(\lambda +i0)}))$ exists in ${\mathfrak S}_1({\mathcal {G}})$ for a.e. $\lambda \in {\mathbb {R}}$.

### Corollary 4.2

Let *A* and *B* be self-adjoint operators in a separable Hilbert space ${\mathfrak H}$ and assume that for some $\zeta _0 \in \rho (A)\cap \rho (B)\cap {\mathbb {R}}$ the sign condition

$$\begin{aligned} (A-\zeta _0 I_{{\mathfrak H}})^{-1}\ge (B-\zeta _0 I_{{\mathfrak H}})^{-1} \end{aligned}$$

holds. Assume that the closed symmetric operator $S=A\cap B$ in (4.1) is densely defined and let $\{{\mathcal {G}},\Gamma _0,\Gamma _1\}$ be a quasi boundary triple with $\gamma$-field $\gamma$ and Weyl function *M* such that (4.2), and hence also (4.3), hold. Assume that $M(z_1)$, $M(z_2)^{-1}$ are bounded (not necessarily everywhere defined ) operators in ${\mathcal {G}}$ for some $z_1,z_2\in \rho (A)$ and that $\overline{\gamma (z_0)}\in {\mathfrak S}_2({\mathcal {G}},{\mathfrak H})$ for some $z_0\in \rho (A)$. Then the following assertions (i)–(iii) hold:

(i) The difference of the resolvents of *A* and *B* is a trace class operator, that is,
  $$\begin{aligned} \big [(B - z I_{{\mathfrak H}})^{-1}-(A - z I_{{\mathfrak H}})^{-1}\big ] \in {\mathfrak S}_1({\mathfrak H}) \end{aligned}$$
  holds for all $z\in \rho (A)\cap \rho (B)$.
(ii) $\mathrm {Im}\big (\log \big (\overline{M(z)}\big )\big )\in {\mathfrak S}_1({\mathcal {G}})$ for all $z\in {\mathbb {C}}\backslash {\mathbb {R}}$ and the limit
  $$\begin{aligned} \mathrm {Im}\big (\log \big (\overline{M(\lambda +i 0)}\big )\big ):=\lim _{\varepsilon \downarrow 0}\mathrm {Im}\big (\log \big (\overline{M(\lambda +i\varepsilon )}\big )\big ) \end{aligned}$$
  exists for a.e. $\lambda \in {\mathbb {R}}$ in ${\mathfrak S}_1({\mathcal {G}})$.
(iii) The function
  4.24 $$\begin{aligned} \xi (\lambda )=\pi ^{-1} {{\mathrm{tr}}}_{{\mathcal {G}}}\bigl (\mathrm {Im}\big (\log \big (\overline{M(\lambda + i0)}\big )\big )\bigr ) \quad \text { for a.e.}~\lambda \in {\mathbb {R}}, \end{aligned}$$
  is a spectral shift function for the pair $\{A,B\}$ such that $\xi (\lambda )=0$ in an open neighborhood of $\zeta _0$ and the trace formula
  $$\begin{aligned} {{\mathrm{tr}}}_{{\mathfrak H}}\bigl ( (B - z I_{{\mathfrak H}})^{-1}-(A - z I_{{\mathfrak H}})^{-1}\bigr ) = - \int _{\mathbb {R}}\frac{\xi (\lambda )\, d\lambda }{(\lambda - z)^{2}} \end{aligned}$$
  is valid for all $z \in \rho (A)\cap \rho (B)$.

### Proof

The assumption $\overline{\gamma (z_0)}\in {\mathfrak S}_2({\mathcal {G}},{\mathfrak H})$ for some $z_0\in \rho (A)$ implies $\overline{\gamma (z)}\in {\mathfrak S}_2({\mathcal {G}},{\mathfrak H})$ for all $z \in \rho (A)$ by (2.5) and hence also $\gamma (z)^*\in {\mathfrak S}_2({\mathfrak H},{\mathcal {G}})$ for all $z \in \rho (A)$. Since $M(z)^{-1}$ is bounded for all $z \in \rho (A)\cap \rho (B)$ (see (2.8)), conditions (4.5)–(4.6) in Theorem 4.1 are satisfied for $k=0$ and all $z\in \rho (A)\cap \rho (B)$. Furthermore,

$$\begin{aligned} \frac{d}{dz}\overline{M(z)}=\gamma (z)^*\overline{\gamma (z)}\in {\mathfrak S}_1({\mathcal {G}}) \end{aligned}$$

by (2.12) and hence (4.7) holds for $k=0$. In particular, by Lemma 3.5 we have

$$\begin{aligned} \frac{d}{dz} \log \big (\overline{M(z)}\big )\in {\mathfrak S}_1({\mathcal {G}}). \end{aligned}$$

In Step 3 of the proof of Theorem 4.1 we can now apply Proposition 3.4 under the assumption (3.25), so that (3.26) holds with $N(\lambda +i0)$ replaced by $\overline{M(\lambda +i0)}$. Now the assertions (i)–(iii) in Corollary 4.2 follow from Theorem 4.1 and Proposition 3.4. $\square$

In the next step we replace the sign condition (4.4) in the assumptions in Theorem 4.1 by some weaker comparability condition. Again, let *A* and *B* be self-adjoint operators in a separable Hilbert space ${\mathfrak H}$ and assume that there exists a self-adjoint operator *C* in ${\mathfrak H}$ such that

4.25 $$\begin{aligned} (C-\zeta _A I_{{\mathfrak H}})^{-1}\ge (A-\zeta _A I_{{\mathfrak H}})^{-1}\quad \text { and } \quad (C-\zeta _B I_{{\mathfrak H}})^{-1}\ge (B-\zeta _B I_{{\mathfrak H}})^{-1} \end{aligned}$$

for some $\zeta _A\in \rho (A)\cap \rho (C)\cap {\mathbb {R}}$ and some $\zeta _B\in \rho (B)\cap \rho (C)\cap {\mathbb {R}}$, respectively. Assume that the closed symmetric operators $S_A=A\cap C$ and $S_B=B\cap C$ are both densely defined and choose quasi boundary triples $\{{\mathcal {G}}_A,\Gamma _0^A,\Gamma _1^A\}$ and $\{{\mathcal {G}}_B,\Gamma _0^B,\Gamma _1^B\}$ with $\gamma$-fields $\gamma _A,\gamma _B$ and Weyl functions $M_A$, $M_B$ for

$$\begin{aligned} T_A=S_A^*\upharpoonright \bigl ({{\mathrm{dom}}}(A)+{{\mathrm{dom}}}(C)\bigr )\quad \text { and } \quad T_B=S_B^*\upharpoonright \bigl ({{\mathrm{dom}}}(B)+{{\mathrm{dom}}}(C)\bigr ) \end{aligned}$$

such that

4.26 $$\begin{aligned} C=T_A\upharpoonright \ker (\Gamma _0^A)=T_B\upharpoonright \ker (\Gamma _0^B), \end{aligned}$$

and

4.27 $$\begin{aligned} A=T_A\upharpoonright \ker (\Gamma _1^A)\quad \text { and } \quad B=T_B\upharpoonright \ker (\Gamma _1^B), \end{aligned}$$

(cf. Proposition 2.4). Next, assume that for some $k \in {\mathbb {N}}_0$, the conditions in Theorem 4.1 are satisfied for the $\gamma$-fields $\gamma _A,\gamma _B$ and the Weyl functions $M_A$, $M_B$. Then the difference of the $2k+1$-th powers of the resolvents of *A* and *C*, and *B* and *C* are trace class operators, and for orthonormal bases $(\varphi _j)_{j \in J}$ in ${\mathcal {G}}_A$ and $(\psi _{\ell })_{\ell \in L}$ in ${\mathcal {G}}_B$ ($J, L \subseteq {\mathbb {N}}$ appropriate index sets),

4.28 $$\begin{aligned} \begin{aligned} \xi _A(\lambda )&=\sum _{j \in J}\lim _{\varepsilon \downarrow 0} \pi ^{-1}\bigl (\mathrm {Im}\big (\log \big (\overline{M_A(\lambda +i\varepsilon )}\big )\big )\varphi _j,\varphi _j\bigr )_{{\mathcal {G}}_A}\quad \text { for a.e.}~\lambda \in {\mathbb {R}},\\ \xi _B(\lambda )&=\sum _{\ell \in L}\lim _{\varepsilon \downarrow 0} \pi ^{-1}\bigl (\mathrm {Im}\big (\log \big (\overline{M_B(\lambda +i\varepsilon )}\big )\big ) \psi _{\ell },\psi _{\ell }\bigr )_{{\mathcal {G}}_B}\quad \text { for a.e.}~\lambda \in {\mathbb {R}}, \end{aligned} \end{aligned}$$

are spectral shift functions for the pairs $\{C,A\}$ and $\{C,B\}$, respectively. It follows for $z \in \rho (A)\cap \rho (B)\cap \rho (C)$ that

$$\begin{aligned} {{\mathrm{tr}}}_{{\mathfrak H}}\bigl ( (B - z I_{{\mathfrak H}})^{-(2k+1)}-(A - z I_{{\mathfrak H}})^{-(2k+1)}\bigr ) = - (2k+1) \int _{\mathbb {R}}\frac{[\xi _B(\lambda ) - \xi _A(\lambda )] \, d\lambda }{(\lambda - z)^{2k+2}} \end{aligned}$$

and $$\int _{\mathbb {R}}\frac{\vert \xi _B(\lambda )-\xi _A(\lambda )\vert \, d\lambda }{(1+\vert \lambda \vert )^{2m+2}} < \infty $$. Therefore,

4.29 $$\begin{aligned} \xi (\lambda ) = \xi _B(\lambda )-\xi _A(\lambda ) \quad \text { for a.e.}~\lambda \in {\mathbb {R}}, \end{aligned}$$

is a spectral shift function for the pair $\{A,B\}$, and in the special case where ${\mathcal {G}}_A = {\mathcal {G}}_B := {\mathcal {G}}$ and $(\varphi _j)_{j \in J}$ is an orthonormal basis in ${\mathcal {G}}$, one infers that

4.30 $$\begin{aligned} \xi (\lambda )&=\sum _{j \in J}\lim _{\varepsilon \downarrow 0}\pi ^{-1} \Bigl (\bigl (\mathrm {Im}\bigl ( \log \big (\overline{M_B(\lambda +i\varepsilon )}\big ) - \log \big (\overline{M_A(\lambda + i \varepsilon )}\big )\bigr ) \varphi _j,\varphi _j\Bigr )_{\mathcal {G}}\nonumber \\&\quad \text {for a.e. } \lambda \in {\mathbb {R}}. \end{aligned}$$

We emphasize that in contrast to the spectral shift function in Theorem 4.1, the spectral shift function $\xi$ in (4.29) and (4.30) is not necessarily nonnegative.

## Elliptic differential operators with Robin boundary conditions

In this section we consider a uniformly elliptic formally symmetric second-order differential expression ${\mathcal {L}}$ on a bounded or unbounded domain in ${\mathbb {R}}^n$ with compact boundary, and we determine a spectral shift function for a pair $\{A_{\beta _0},A_{\beta _1}\}$ consisting of two self-adjoint Robin-realizations of ${\mathcal {L}}$. We shall assume throughout this section that the following hypothesis holds.

### Hypothesis 5.1

Let $n \in {\mathbb {N}}$, $n \ge 2$, and $\Omega \subseteq {\mathbb {R}}^n$ be nonempty and open such that its boundary $\partial \Omega$ is nonempty, $C^\infty$-smooth, and compact. Consider the differential expression

5.1 $$\begin{aligned} {\mathcal {L}}= -\sum _{j,k=1}^n \left( \frac{\partial }{\partial x_j} \Bigl (a_{jk} \frac{\partial }{\partial x_k}\Bigr )\right) + a \end{aligned}$$

on $\Omega$, where the real-valued coefficients $a_{jk}\in C^\infty (\overline{\Omega })$ satisfy $a_{jk}(x)=a_{kj}(x)$ for all $x\in \overline{\Omega }$ and $j,k=1,\dots ,n$, their first partial derivatives are bounded in $\overline{\Omega }$, and $a\in C^\infty (\overline{\Omega })$ is a real-valued, bounded, measurable function. Furthermore, it is assumed that ${\mathcal {L}}$ is uniformly elliptic on $\overline{\Omega }$, that is, for some $C>0$,

5.2 $$\begin{aligned} \sum _{j,k=1}^n a_{jk}(x)\xi _j\xi _k\ge C\sum _{k=1}^n\xi _k^2 \end{aligned}$$

holds for all $\xi =(\xi _1,\dots ,\xi _n)^\top \in {\mathbb {R}}^n$ and $x\in \overline{\Omega }$.

We briefly recall the definition and some mapping properties of the Dirichlet and (oblique) Neumann trace maps associated with the differential expression ${\mathcal {L}}$. For a function $f\in C^\infty (\overline{\Omega })$ we denote its trace by $\gamma _D f=f\vert _{\partial \Omega }$ and we set

$$\begin{aligned} \gamma _\nu f= \sum _{j,k=1}^n a_{jk} \mathfrak n_j \frac{\partial f}{\partial x_k} \Bigl |_{\partial \Omega },\quad f\in C^\infty (\overline{\Omega }), \end{aligned}$$

where $\mathfrak n(x)=(\mathfrak n_1(x),\dots , \mathfrak n_n(x))^\top$ is the unit normal vector at $x\in \partial \Omega$ pointing out of the domain $\Omega$. Let $C_0^\infty (\overline{\Omega }):=\{h\vert _{\overline{\Omega }} \, | \,h\in C_0^\infty ({\mathbb {R}}^n)\}$ and recall that the mapping $C_0^\infty (\overline{\Omega })\ni f\mapsto \{\gamma _D f,\gamma _\nu f\}$ can be extended to a continuous surjective mapping

5.3 $$\begin{aligned} H^2(\Omega )\ni f\mapsto \left\{ \gamma _D f,\gamma _\nu f\right\} \in H^{3/2}(\partial \Omega )\times H^{1/2}(\partial \Omega ), \end{aligned}$$

and that Green’s second identity

5.4 $$\begin{aligned} ({\mathcal {L}}f,g)_{L^2(\Omega )}-(f,{\mathcal {L}}g)_{L^2(\Omega )}=(\gamma _D f,\gamma _\nu g)_{L^2(\partial \Omega )}-(\gamma _\nu f,\gamma _D g)_{L^2(\partial \Omega )} \end{aligned}$$

is valid for all $f,g\in H^2(\Omega )$; cf. [53]. We will also use the fact that

5.5 $$\begin{aligned} \gamma _D f\in H^{k-1/2}(\partial \Omega )\quad \text { for all } \, f\in H^k(\Omega ), \; k \in {\mathbb {N}}. \end{aligned}$$

The following lemma is a variant of [5, Lemma 4.7]; it will be useful for the ${\mathfrak S}_p$-estimates in this and the next section.

### Lemma 5.2

Let $\Omega \subseteq {\mathbb {R}}^n$ be as in Hypothesis 5.1, let $X\in {\mathcal {L}}\big (L^2(\Omega ),H^t(\partial \Omega )\big )$, and assume that ${{\mathrm{ran}}}(X)\subseteq H^s(\partial \Omega )$ for some $s>t\ge 0$. Then *X* is compact and

$$\begin{aligned} X\in {\mathfrak S}_r\bigl (L^2(\Omega ),H^t(\partial \Omega )\bigr )\quad \text { for all } \, r> (n-1)/(s-t). \end{aligned}$$

Assume that $\beta _0\in C^1(\partial \Omega )$ and $\beta _1\in C^1(\partial \Omega )$ are real-valued functions. For $p=0,1$ we consider the elliptic differential operators in $L^2(\Omega )$,

5.6 $$\begin{aligned} A_{\beta _p} f={\mathcal {L}}f,\quad {{\mathrm{dom}}}(A_{\beta _p}) = \bigl \{f\in H^2(\Omega ) \, \big | \,\beta _p \gamma _D f=\gamma _\nu f\bigr \}, \end{aligned}$$

which correspond to the densely defined, closed, semibounded quadratic forms

5.7 $$\begin{aligned} \mathfrak a_{\beta _p}[f,g]=\sum _{j,k=1}^n \left( a_{jk} \frac{\partial f}{\partial x_k},\frac{\partial g}{\partial x_j}\right) _{L^2(\Omega )}+(af,g)_{L^2(\Omega )} - (\beta _p\gamma _D f,\gamma _D g)_{L^2(\partial \Omega )} \end{aligned}$$

defined on $H^1(\Omega )\times H^1(\Omega )$. Both operators $A_{\beta _0}$ and $A_{\beta _1}$ are self-adjoint in $L^2(\Omega )$ and semibounded from below. For $\beta \in {\mathbb {R}}$ we shall also make use of the self-adjoint Robin realization

5.8 $$\begin{aligned} A_\beta f={\mathcal {L}}f,\quad {{\mathrm{dom}}}(A_\beta ) = \bigl \{f\in H^2(\Omega ) \, \big | \,\beta \gamma _D f=\gamma _\nu f\bigr \}, \end{aligned}$$

which corresponds to the densely defined, closed, semibounded quadratic form

5.9 $$\begin{aligned} \mathfrak a_\beta [f,g]=\sum _{j,k=1}^n \left( a_{jk} \frac{\partial f}{\partial x_k},\frac{\partial g}{\partial x_j}\right) _{L^2(\Omega )}+(af,g)_{L^2(\Omega )} - (\beta \gamma _D f,\gamma _D g)_{L^2(\partial \Omega )} \end{aligned}$$

on $$H^1(\Omega )\times H^1(\Omega )$$.

Next, we define the Neumann-to-Dirichlet map associated to ${\mathcal {L}}$ as a densely defined operator in $L^2(\partial \Omega )$. First one notes that for $\beta _0=0$ in (5.6) (or $\beta =0$ in (5.8)) one obtains

5.10 $$\begin{aligned} A_N:=A_0=A_{\beta _0}, \end{aligned}$$

where $A_N$ denotes the self-adjoint Neumann realization of ${\mathcal {L}}$ in $L^2(\Omega )$. One recalls that for $\varphi \in H^{1/2}(\partial \Omega )$ and $z \in \rho (A_N)$, the boundary value problem

5.11 $$\begin{aligned} {\mathcal {L}}f_z = z f_z ,\quad \gamma _\nu f_z =\varphi , \end{aligned}$$

admits a unique solution $f_z \in H^2(\Omega )$; this follows, for instance, from (5.3) and $z \in \rho (A_N)$. The corresponding solution operator is denoted by

5.12 $$\begin{aligned} P_\nu (z):L^2(\partial \Omega ) \rightarrow L^2(\Omega ),\quad \varphi \mapsto f_z , \end{aligned}$$

and it is clear that ${{\mathrm{dom}}}(P_\nu (z))=H^{1/2}(\partial \Omega )$ and ${{\mathrm{ran}}}(P_\nu (z))\subseteq H^2(\Omega )$. For $z \in \rho (A_N)$ the *Neumann-to-Dirichlet map* associated to ${\mathcal {L}}$ is defined as

5.13 $$\begin{aligned} {\mathcal {N}}(z):L^2(\partial \Omega ) \rightarrow L^2(\partial \Omega ),\quad \varphi \mapsto \gamma _D P_\nu (z)\varphi ; \end{aligned}$$

it maps the (oblique) Neumann boundary values $\gamma _\nu f_z$ of solutions $f_z \in H^2(\Omega )$ of (5.11) onto the Dirichlet boundary values $\gamma _D f_z$. It follows from the properties of the trace maps that

5.14 $$\begin{aligned} {{\mathrm{dom}}}({\mathcal {N}}(z))=H^{1/2}(\partial \Omega )\quad \text { and } \quad {{\mathrm{ran}}}({\mathcal {N}}(z))\subseteq H^{3/2}(\partial \Omega ). \end{aligned}$$

In the next theorem a spectral shift function for the pair $\{A_{\beta _0},A_{\beta _1}\}$ is expressed in terms of the limits of the Neumann-to-Dirichlet map ${\mathcal {N}}(z)$ and the functions $\beta _0$ and $\beta _1$ in the boundary conditions of the Robin realizations $A_{\beta _0}$ and $A_{\beta _1}$. We mention that the trace class condition for the difference of the $2k+1$-th powers of the resolvents was shown for $k=0$ in [4, 34] and for $k \in {\mathbb {N}}$ in [6].

### Theorem 5.3

Assume Hypothesis 5.1, let $A_{\beta _0}$ and $A_{\beta _1}$ be the self-adjoint Robin realizations of ${\mathcal {L}}$ in $L^2(\Omega )$ in (5.6), let $\beta \in {\mathbb {R}}$ such that $\beta _p(x)<\beta$ for all $x\in \partial \Omega$ and $p=0,1$ and let $A_\beta$ be the self-adjoint Robin realizations of ${\mathcal {L}}$ in (5.8). Furthermore, let

$$\begin{aligned} {\mathcal {M}}_p(z)= (\beta -\beta _p)^{-1} \bigl (\beta _p\overline{{\mathcal {N}}(z)}-I_{L^2(\partial \Omega )}\bigr )\bigl (\beta \overline{{\mathcal {N}}(z)}-I_{L^2(\partial \Omega )}\bigr )^{-1}, \quad z\in {\mathbb {C}}\backslash {\mathbb {R}}, \; j=1,2, \end{aligned}$$

where $\overline{{\mathcal {N}}(z)}$ denotes the closure in $L^2(\partial \Omega )$ of the Neumann-to-Dirichlet map associated with ${\mathcal {L}}$ in (5.13). Then the following assertions (*i*) and (*ii*) hold for $k \in {\mathbb {N}}_0$ such that $k\ge (n-3)/4$:

(i) The difference of the $2k+1$th-powers of the resolvents of $A_{\beta _0}$ and $A_{\beta _1}$ is a trace class operator, that is,
  $$\begin{aligned} \big [(A_{\beta _1} - z I_{L^2(\Omega )})^{-(2k+1)} - (A_{\beta _0} - z I_{L^2(\Omega )})^{-(2k+1)}\big ] \in {\mathfrak S}_1\bigl (L^2(\Omega )\bigr ) \end{aligned}$$
  holds for all $z\in \rho (A_{\beta _0})\cap \rho (A_{\beta _1})$.
(ii) For any orthonormal basis $(\varphi _j)_{j \in J}$ in $L^2(\partial \Omega )$ the function
  $$\begin{aligned}&\xi (\lambda ) =\sum _{j \in J} \lim _{\varepsilon \downarrow 0}\pi ^{-1} \Bigl (\bigl (\mathrm {Im}\bigl ( \log ({\mathcal {M}}_1(\lambda +i\varepsilon )) - \log ({\mathcal {M}}_0(\lambda + i \varepsilon ))\bigr )\bigr ) \varphi _j,\varphi _j\Bigr )_{L^2(\partial \Omega )} \end{aligned}$$
  for a.e. $\lambda \in {\mathbb {R}}$, is a spectral shift function for the pair $\{A_{\beta _0},A_{\beta _1}\}$ such that $\xi (\lambda )=0$ for $\lambda < \min (\sigma (A_\beta ))$ and the trace formula
  $$\begin{aligned} {{\mathrm{tr}}}_{L^2(\Omega )}\bigl ( (A_{\beta _1} - z I_{L^2(\Omega )})^{-(2k+1)} - (A_{\beta _0} - z I_{L^2(\Omega )})^{-(2k+1)}\bigr ) = - (2k+1) \int _{\mathbb {R}}\frac{\,\xi (\lambda )\, d\lambda }{(\lambda - z)^{2k+2}} \end{aligned}$$
  is valid for all $z \in \rho (A_{\beta _0})\cap \rho (A_{\beta _1})$.

### Proof

The proof of Theorem 5.3 consists of three steps. In the first step we construct a suitable quasi boundary triple such that the self-adjoint operators $A_\beta$ and $A_{\beta _1}$ correspond to the kernels of the boundary mappings $\Gamma _0$ and $\Gamma _1$, and in the second and third step we show that the pair $\{A_{\beta },A_{\beta _1}\}$ and the $\gamma$-field and Weyl function satisfy the assumptions in Theorem 4.1. The same reasoning applies to the pair $\{A_{\beta },A_{\beta _0}\}$, and hence Theorem 4.1 can be applied to both pairs $\{A_{\beta },A_{\beta _1}\}$ and $\{A_{\beta },A_{\beta _0}\}$, which together with the considerations at the end of Sect. 4 yield the assertions in Theorem 5.3.

*Step 1*  The basic techniques in this step have been used in a similar framework, for instance, in [2, 3, 5, 8]. We consider the closed symmetric operator $S=A_{\beta }\cap A_{\beta _1}$, which is given by

5.15 $$\begin{aligned} Sf={\mathcal {L}}f,\quad {{\mathrm{dom}}}(S)=\bigl \{f\in H^2(\Omega ) \, \big | \, \gamma _D f=\gamma _\nu f=0\bigr \}, \end{aligned}$$

where we have used that $\beta -\beta _1(x)\not =0$ for all $x\in \partial \Omega$. In this step we check that the operator

5.16 $$\begin{aligned} Tf={\mathcal {L}}f,\quad {{\mathrm{dom}}}(T)=H^2(\Omega ), \end{aligned}$$

satisfies $\overline{T}=S^*$ and that $\big \{L^2(\partial \Omega ),\Gamma _0,\Gamma _1\big \}$, where

5.17 $$\begin{aligned} \Gamma _0 f=\beta \gamma _D f - \gamma _\nu f,\quad \Gamma _1 f= (\beta -\beta _1)^{-1} \bigl (\beta _1 \gamma _D f - \gamma _\nu f\bigr ),\quad f\in {{\mathrm{dom}}}(T), \end{aligned}$$

is a quasi boundary triple for $T\subset S^*$ such that

5.18 $$\begin{aligned} A_{\beta }=T\upharpoonright \ker (\Gamma _0)\, \text { and } \, A_{\beta _1}=T\upharpoonright \ker (\Gamma _1), \end{aligned}$$

and for all $z \in \rho (A_{\beta })\cap \rho (A_N)$, where $A_N$ is the self-adjoint Neumann realization in (5.10), the corresponding $\gamma$-field $\gamma$ and Weyl function *M* in $L^2(\partial \Omega )$ are given by

5.19 $$\begin{aligned} \gamma (z)=P_\nu (z)\bigl (\beta {\mathcal {N}}(z)-I_{L^2(\partial \Omega )}\bigr )^{-1},\quad {{\mathrm{dom}}}(\gamma (z))=H^{1/2}(\partial \Omega ), \end{aligned}$$

and

5.20 $$\begin{aligned} \begin{aligned}&M(z) = (\beta -\beta _1)^{-1} \bigl (\beta _1{\mathcal {N}}(z)-I_{L^2(\partial \Omega )}\bigr )\bigl (\beta {\mathcal {N}}(z)-I_{L^2(\partial \Omega )}\bigr )^{-1},\\&{{\mathrm{dom}}}(M(z)) = H^{1/2}(\partial \Omega ). \end{aligned} \end{aligned}$$

We will use Theorem 2.2 for this purpose. For $f,g\in {{\mathrm{dom}}}(T)=H^2(\Omega )$ one obtains with the help of Green’s identity (5.4),

$$\begin{aligned} \begin{aligned}&(\Gamma _1 f,\Gamma _0 g)_{L^2(\partial \Omega )}-(\Gamma _0 f,\Gamma _1 g)_{L^2(\partial \Omega )}\\&\quad =\bigl ((\beta -\beta _1)^{-1}(\beta _1 \gamma _D f - \gamma _\nu f),\beta \gamma _D g - \gamma _\nu g\bigr )_{L^2(\partial \Omega )}\\&\qquad - \bigl (\beta \gamma _D f - \gamma _\nu f,(\beta -\beta _1)^{-1}(\beta _1 \gamma _D g - \gamma _\nu g)\bigr )_{L^2(\partial \Omega )}\\&\quad =\bigl (\gamma _D f,\gamma _\nu g\bigr )_{L^2(\partial \Omega )}-\bigl (\gamma _\nu f,\gamma _D g\bigr )_{L^2(\partial \Omega )}=({\mathcal {L}}f,g)_{L^2(\Omega )}-(f,{\mathcal {L}}g)_{L^2(\Omega )}\\&\quad =(Tf,g)_{L^2(\Omega )}-(f,Tg)_{L^2(\Omega )}, \end{aligned} \end{aligned}$$

and hence condition (*i*) in Theorem 2.2 holds. Since

5.21 $$\begin{aligned} \begin{pmatrix}\Gamma _0 f\\ \Gamma _1 f\end{pmatrix}=\begin{pmatrix} \beta &{} -I_{L^2(\partial \Omega )} \\ \beta _1(\beta -\beta _1)^{-1} &{} - (\beta -\beta _1)^{-1} \end{pmatrix} \begin{pmatrix}\gamma _D f\\ \gamma _\nu f\end{pmatrix},\quad f\in {{\mathrm{dom}}}(T), \end{aligned}$$

and the $2\times 2$ operator matrix in (5.21) is an isomorphism in $L^2(\partial \Omega )\times L^2(\partial \Omega )$, it follows from (5.3) that ${{\mathrm{ran}}}(\Gamma _0,\Gamma _1)^\top$ is dense in $L^2(\partial \Omega )\times L^2(\partial \Omega )$. It is easy to see that $\ker (\Gamma _0)\cap \ker (\Gamma _1)$ is dense in $L^2(\Omega )$. Moreover, (5.18) is clear from the definition of *T* and the boundary maps in (5.17). Hence also conditions (*ii*) and (*iii*) in Theorem 2.2 are satisfied, and from (5.15)–(5.17) one obtains $S=T\upharpoonright (\ker (\Gamma _0)\cap \ker (\Gamma _1))$. Thus Theorem 2.2 yields $\overline{T}=S^*$ and that $\{L^2(\partial \Omega ),\Gamma _0,\Gamma _1\}$ is a quasi boundary triple for $S^*$ such that (5.18) holds.

It remains to show the explicit form of the corresponding $\gamma$-field and Weyl function *M* in (5.19) and (5.20), respectively. First of all it follows from (5.3) and the definition of $\Gamma _0$ in (5.17) that

$$\begin{aligned} H^{1/2}(\partial \Omega )={{\mathrm{ran}}}(\Gamma _0)={{\mathrm{dom}}}(\gamma (z))={{\mathrm{dom}}}(M(z)),\quad z \in \rho (A_{\beta }). \end{aligned}$$

One notes that for $z \in \rho (A_N)$ and $f_z \in \ker (T - z I_{L^2(\Omega )})$ one has ${\mathcal {N}}(z)\gamma _\nu f_z =\gamma _D f_z$ according to (5.13), and hence

5.22 $$\begin{aligned} \bigl (\beta {\mathcal {N}}(z)-I_{L^2(\partial \Omega )}\bigr )\gamma _\nu f_z = \beta \gamma _D f_z -\gamma _\nu f_z =\Gamma _0 f_z, \end{aligned}$$

and

5.23 $$\begin{aligned} (\beta -\beta _1)^{-1}\bigl (\beta _1{\mathcal {N}}(z)-I_{L^2(\partial \Omega )}\bigr )\gamma _\nu f_z = (\beta -\beta _1)^{-1}\bigl (\beta _1\gamma _D f_z -\gamma _\nu f_z \bigr )=\Gamma _1 f_z,\quad \end{aligned}$$

by (5.17). The relation (5.22) also shows that $\ker (\beta {\mathcal {N}}(z)-I_{L^2(\partial \Omega )})=\{0\}$ for $z \in \rho (A_{\beta })\cap \rho (A_N)$ and hence

5.24 $$\begin{aligned} \gamma _\nu f_z =\bigl (\beta {\mathcal {N}}(z)-I_{L^2(\partial \Omega )}\bigr )^{-1}\Gamma _0 f_z . \end{aligned}$$

From this and (5.12) it follows that the $\gamma$-field corresponding to $\{L^2(\partial \Omega ),\Gamma _0,\Gamma _1\}$ has the form (5.19). One also concludes from (5.24) and (5.23) that

$$\begin{aligned} (\beta -\beta _1)^{-1}\bigl (\beta _1{\mathcal {N}}(z)-I_{L^2(\partial \Omega )}\bigr )\bigl (\beta {\mathcal {N}}(z)-I_{L^2(\partial \Omega )}\bigr )^{-1}\Gamma _0 f_z =\Gamma _1 f_z \end{aligned}$$

holds for all $f_z \in \ker (T - z I_{L^2(\Omega )})$ and $z \in \rho (A_{\beta })\cap \rho (A_N)$. Thus the Weyl function corresponding to the quasi boundary triple $\{L^2(\partial \Omega ),\Gamma _0,\Gamma _1\}$ has the form (5.20).

*Step 2*  In this step we verify that the pair $\{A_{\beta },A_{\beta _1}\}$ satisfies the sign condition (4.4) and that the values of Weyl function and its inverse are bounded operators; see the assumptions of Theorem 4.1.

The assumption $\beta >\beta _1(x)$ shows that the semibounded quadratic forms $\mathfrak a_{\beta }$ and $\mathfrak a_{\beta _1}$ in (5.7) and (5.9) corresponding to $A_{\beta }$ and $A_{\beta _1}$ satisfy the inequality $\mathfrak a_{\beta }\le \mathfrak a_{\beta _1}$. Hence $\min (\sigma (A_{\beta }))\le \min (\sigma (A_{\beta _1}))$ and for $\zeta < \min (\sigma (A_{\beta }))$ the forms $\mathfrak a_{\beta }-\zeta$ and $\mathfrak a_{\beta _1}-\zeta$ are both nonnegative, satisfy the inequality $\mathfrak a_{\beta }-\zeta \le \mathfrak a_{\beta _1}-\zeta$, and hence the resolvents of the corresponding nonnegative self-adjoint operators $A_{\beta }-\zeta I_{L^2(\Omega )}$ and $A_{\beta _1}-\zeta I_{L^2(\Omega )}$ satisfy the inequality

$$\begin{aligned} \left( A_{\beta }-\zeta I_{L^2(\Omega )}\right) ^{-1}\ge \left( A_{\beta _1}-\zeta I_{L^2(\Omega )}\right) ^{-1}, \quad \zeta < \min (\sigma (A_{\beta })) \end{aligned}$$

(see, e.g., [41, Chapter VI, $\S$ 2.6] or [15, Chapter 10, $\S$2-Theorem 6]). Thus the sign condition (4.4) in the assumptions of Theorem 4.1 holds.

Next we prove that

5.25 $$\begin{aligned} \begin{aligned} M(z_1)&=(\beta -\beta _1)^{-1}\bigl (\beta _1{\mathcal {N}}(z_1)-I_{L^2(\partial \Omega )}\bigr )\bigl (\beta {\mathcal {N}}(z_1)-I_{L^2(\partial \Omega )}\bigr )^{-1},\\ M(z_2)^{-1}&=\bigl (\beta {\mathcal {N}}(z_2)-I_{L^2(\partial \Omega )}\bigr )\bigl (\beta _1{\mathcal {N}}(z_2)-I_{L^2(\partial \Omega )}\bigr )^{-1}(\beta -\beta _1), \end{aligned} \end{aligned}$$

are bounded operators for some $z_1,z_2\in {\mathbb {C}}\backslash {\mathbb {R}}$. According to [5, Lemma 4.4] the closure $\overline{{\mathcal {N}}(z)}$, $z\in {\mathbb {C}}\backslash {\mathbb {R}}$, of the Neumann-to-Dirichlet map in (5.13) in $L^2(\partial \Omega )$ is compact, and hence $\beta {\mathcal {N}}(z)-I_{L^2(\partial \Omega )}$ and $\beta _1{\mathcal {N}}(z)-I_{L^2(\partial \Omega )}$ are densely defined bounded operators in $L^2(\partial \Omega )$, and for $z\in {\mathbb {C}}\backslash {\mathbb {R}}$ their closures are

5.26 $$\begin{aligned} \big [\beta \overline{{\mathcal {N}}(z)}-I_{L^2(\partial \Omega )}\big ] \in {\mathcal {L}}\bigl (L^2(\partial \Omega )\bigr ) \quad \text { and } \quad \big [\beta _1\overline{{\mathcal {N}}(z)}-I_{L^2(\partial \Omega )}\big ] \in {\mathcal {L}}\bigl (L^2(\partial \Omega )\bigr ).\qquad \end{aligned}$$

In order to see that

$$\begin{aligned} Q(z):=\bigl (\beta {\mathcal {N}}(z)-I_{L^2(\partial \Omega )}\bigr )^{-1}\quad \text { and } \quad Q_1(z):=\bigl (\beta _1{\mathcal {N}}(z)-I_{L^2(\partial \Omega )}\bigr )^{-1} \end{aligned}$$

are bounded for $z\in {\mathbb {C}}\backslash {\mathbb {R}}$ we argue in a similar way as in the proof of [5, Lemma 4.4]: First, one notes that ${\mathcal {N}}(z)\subseteq {\mathcal {N}}({\overline{z}})^*$, $z\in {\mathbb {C}}\backslash {\mathbb {R}}$, holds by (5.4), and this yields that also $Q(z)\subseteq Q({\overline{z}})^*$, $z\in {\mathbb {C}}\backslash {\mathbb {R}}$. Hence the operator *Q*(*z*) is closable in $L^2(\partial \Omega )$. Moreover, as *Q*(*z*) is defined on $H^{1/2}(\partial \Omega )$ and maps into $H^{1/2}(\partial \Omega )$, it follows that *Q*(*z*) is a closed operator in $H^{1/2}(\partial \Omega )$, and hence

5.27 $$\begin{aligned} Q(z):H^{1/2}(\partial \Omega )\rightarrow H^{1/2}(\partial \Omega ),\quad \varphi \mapsto Q(z)\varphi , \end{aligned}$$

is bounded. Therefore, the dual operator

5.28 $$\begin{aligned} Q(z)^\prime :H^{-1/2}(\partial \Omega )\rightarrow H^{-1/2}(\partial \Omega ),\quad \psi \mapsto Q(z)^\prime \psi , \end{aligned}$$

where $(Q(z)^\prime \psi )(\varphi )=\psi (Q(z)\varphi )$, $\varphi \in H^{1/2}(\partial \Omega )$, is also bounded. One verifies that $Q({\overline{z}})^\prime$ is an extension of *Q*(*z*) and hence by interpolation and (5.27) and (5.28), the restriction

$$\begin{aligned} Q({\overline{z}})^\prime \upharpoonright _{L^2(\partial \Omega )}:L^2(\partial \Omega )\rightarrow L^2(\partial \Omega ),\quad \phi \mapsto Q({\overline{z}})^\prime \phi , \end{aligned}$$

of $Q({\overline{z}})^\prime$ onto $L^2(\partial \Omega )$ is a bounded operator in $L^2(\partial \Omega )$ and an extension of *Q*(*z*). Hence for all $z\in {\mathbb {C}}\backslash {\mathbb {R}}$ the operator *Q*(*z*) is bounded in $L^2(\partial \Omega )$ and its closure is

5.29 $$\begin{aligned} \overline{Q(z)}= \bigl (\beta \overline{{\mathcal {N}}(z)}-I_{L^2(\partial \Omega )}\bigr )^{-1}\in {\mathcal {L}}\bigl (L^2(\partial \Omega )\bigr ),\quad z\in {\mathbb {C}}\backslash {\mathbb {R}}. \end{aligned}$$

The same reasoning with *Q*(*z*) replaced by $Q_1(z)$ shows that for all $z\in {\mathbb {C}}\backslash {\mathbb {R}}$ the operator $Q_1(z)$ is bounded in $L^2(\partial \Omega )$ and

5.30 $$\begin{aligned} \overline{Q_1(z)}= \bigl (\beta _1\overline{{\mathcal {N}}(z)}-I_{L^2(\partial \Omega )}\bigr )^{-1}\in {\mathcal {L}}\bigl (L^2(\partial \Omega )\bigr ),\quad z\in {\mathbb {C}}\backslash {\mathbb {R}}. \end{aligned}$$

Next, it follows that $M(z_1)$ and $M(z_2)^{-1}$ in (5.25) are bounded in $L^2(\partial \Omega )$ for $z_1,z_2\in {\mathbb {C}}\backslash {\mathbb {R}}$ and the closure of *M*(*z*) is given by

5.31 $$\begin{aligned} \overline{M(z)}= (\beta -\beta _1)^{-1}\bigl (\beta _1\overline{{\mathcal {N}}(z)}-I_{L^2(\partial \Omega )}\bigr )\bigl (\beta \overline{{\mathcal {N}}(z)}-I_{L^2(\partial \Omega )}\bigr )^{-1} \end{aligned}$$

by (5.26) and (5.29). One notes that $\overline{M(z)}={\mathcal {M}}_1(z)$ in the formulation of Theorem 5.3.

*Step 3* In this step we verify that the $\gamma$-field and Weyl function corresponding to the quasi boundary triple $\big \{L^2(\partial \Omega ),\Gamma _0,\Gamma _1\big \}$ in Step 1 satisfy the ${\mathfrak S}_p$-conditions in the assumptions of Theorem 4.1 for dimensions $n \in {\mathbb {N}}$, $n \ge 2$, and $k\ge (n-3)/4$, that is, we verify for all $p,q \in {\mathbb {N}}_0$ and all $z \in \rho (A_{\beta })\cap \rho (A_{\beta _1})$ the conditions

5.32 $$\begin{aligned} \overline{\gamma (z)}^{(p)}\bigl ( M(z)^{-1} \gamma ({\overline{z}})^*\bigr )^{(q)}\in & {} {\mathfrak S}_1\bigl (L^2(\Omega )\bigr ),\quad p+q=2k, \end{aligned}$$

5.33 $$\begin{aligned} \bigl ( M(z)^{-1} \gamma ({\overline{z}})^*\bigr )^{(q)}\overline{\gamma (z)}^{(p)}\in & {} {\mathfrak S}_1\bigl (L^2(\partial \Omega )\bigr ),\quad p+q=2k, \end{aligned}$$

and

5.34 $$\begin{aligned} \frac{d^j}{dz^j} \overline{M (z)}\in {\mathfrak S}_{(2k+1)/j}\bigl (L^2(\partial \Omega )\bigr ),\quad j=1,\dots ,2k+1. \end{aligned}$$

In the following we shall often use the smoothing property

5.35 $$\begin{aligned} \left( A_{\beta } - z I_{L^2(\Omega )}\right) ^{-1} f \in H^{k+2}(\Omega )\quad \text { for all } \, f\in H^k(\Omega ), \; k \in {\mathbb {N}}_0, \end{aligned}$$

of the resolvent of $A_{\beta }$, which follows, for instance, from [59, Theorem 4.18]. One notes that (2.2) and the definition of the boundary map $\Gamma _1$ in (5.17) yield

5.36 $$\begin{aligned} \begin{aligned} \gamma ({\overline{z}})^*f&=\Gamma _1\left( A_{\beta } - z I_{L^2(\Omega )}\right) ^{-1}f\\&=(\beta -\beta _1)^{-1}\bigl (\beta \gamma _D - \gamma _\nu + (\beta _1-\beta )\gamma _D\bigr ) \left( A_{\beta } - z I_{L^2(\Omega )}\right) ^{-1}f\\&=(\beta -\beta _1)^{-1}(\beta \gamma _D - \gamma _\nu )\left( A_{\beta } - z I_{L^2(\Omega )}\right) ^{-1}f - \gamma _D\left( A_{\beta } - z I_{L^2(\Omega )}\right) ^{-1}f\\&=- \gamma _D\left( A_{\beta } - z I_{L^2(\Omega )}\right) ^{-1}f \end{aligned} \end{aligned}$$

for all $z \in \rho (A_\beta )$ and $f\in L^2(\Omega )$. Here we have used in the last step that

$$\begin{aligned} g=\left( A_{\beta } - z I_{L^2(\Omega )}\right) ^{-1}f\in {{\mathrm{dom}}}(A_{\beta }) \end{aligned}$$

satisfies the boundary condition $\beta \gamma _Dg - \gamma _\nu g=0$. It follows from (2.6) and (5.36) that

$$\begin{aligned} \bigl (\gamma ({\overline{z}})^*\bigr )^{(q)}=q! \, \gamma ({\overline{z}})^*\left( A_{\beta } - z I_{L^2(\Omega )}\right) ^{-q} = - q! \, \gamma _D\left( A_{\beta } - z I_{L^2(\Omega )}\right) ^{-(q+1)}, \end{aligned}$$

and hence, ${{\mathrm{ran}}}((\gamma ({\overline{z}})^*)^{(q)})\subset H^{2q+3/2}(\partial \Omega )$ by (5.35) and (5.5). From Lemma 5.2 with $s=2q+(3/2)$ and $t=0$ one concludes that

5.37 $$\begin{aligned} \bigl (\gamma ({\overline{z}})^*\bigr )^{(q)} \in {\mathfrak S}_r\bigl (L^2(\Omega ),L^2(\partial \Omega )\bigr ), \quad r>(n-1)/[2q+(3/2)], \end{aligned}$$

for all $z \in \rho (A_{\beta })$, $q \in {\mathbb {N}}_0$, and hence by (2.6) also

5.38 $$\begin{aligned} \overline{\gamma (z)}^{(p)}\in {\mathfrak S}_r\bigl (L^2(\partial \Omega ),L^2(\Omega )\bigr ), \quad r> (n-1)/[2p+(3/2)], \end{aligned}$$

for all $z \in \rho (A_{\beta })$, $p \in {\mathbb {N}}_0$. Furthermore,

5.39 $$\begin{aligned} \frac{d^j}{dz^j} \overline{M (z)}=j! \, \gamma ({\overline{z}})^* \left( A_{\beta } - z I_{L^2(\Omega )}\right) ^{-(j-1)}\overline{\gamma (z)}, \quad j \in {\mathbb {N}}, \end{aligned}$$

by (2.12) and with the help of (5.36) it follows in the same way as in (5.37) that

$$\begin{aligned} \gamma ({\overline{z}})^*\left( A_{\beta } - z I_{L^2(\Omega )}\right) ^{-(j-1)} = - \gamma _D \left( A_{\beta } - z I_{L^2(\Omega )}\right) ^{-j} \in {\mathfrak S}_x\big (L^2(\Omega ),L^2(\partial \Omega )\big ) \end{aligned}$$

for $x> (n-1)/[2j-(1/2)]$. Moreover, $\overline{\gamma (z)}\in {\mathfrak S}_y(L^2(\partial \Omega ),L^2(\Omega ))$ for $y> 2 (n-1)/3$ by (5.38) and hence it follows from (5.39) and the well-known property $PQ\in {\mathfrak S}_w$ for $P\in {\mathfrak S}_x$, $Q\in {\mathfrak S}_y$, and $x^{-1}+y^{-1}=w^{-1}$, that

5.40 $$\begin{aligned} \frac{d^j}{dz^j} \overline{M(z)}\in {\mathfrak S}_w\bigl (L^2(\partial \Omega \bigr ), \quad w > (n-1)/(2j+1), \quad z \in \rho (A_{\beta }), \quad j \in {\mathbb {N}}.\qquad \end{aligned}$$

One observes that

$$\begin{aligned} \frac{d}{dz} \overline{M(z)}^{-1}= - \overline{M(z)}^{-1}\left( \frac{d}{dz} \overline{M(z)}\right) \overline{M(z)}^{-1}, \quad z \in \rho (A_{\beta })\cap \rho (A_{\beta _1}), \end{aligned}$$

that $\overline{M(z)}^{-1}$ is bounded, and by (5.40) that also

5.41 $$\begin{aligned} \frac{d^j}{dz^j} \overline{M (z)}^{-1}&\in {\mathfrak S}_w\bigl (L^2(\partial \Omega )\bigr ), \quad w > (n-1)/(2j+1),\nonumber \\ z&\in \rho (A_{\beta })\cap \rho (A_{\beta _1}), \; j \in {\mathbb {N}}; \end{aligned}$$

we leave the formal induction step to the reader. Therefore,

$$\begin{aligned} \begin{aligned} \bigl ( M(z)^{-1} \gamma ({\overline{z}})^* \bigr )^{(q)}&=\bigl ( \overline{M(z)}^{-1} \gamma ({\overline{z}})^*\bigr )^{(q)} =\sum _{\begin{array}{c} p+m=q \\ p,m\geqslant 0 \end{array}} \begin{pmatrix} q \\ p \end{pmatrix} \bigl (\overline{M(z)}^{-1}\bigr )^{(p)} \bigl (\gamma ({\overline{z}})^*\bigr )^{(m)}\\&=\overline{M(z)}^{-1} \bigl (\gamma ({\overline{z}})^*\bigr )^{(q)}+ \sum _{\begin{array}{c} p+m=q \\ p > 0, m\ge 0 \end{array}} \begin{pmatrix} q \\ p \end{pmatrix} \bigl (\overline{M(z)}^{-1}\bigr )^{(p)} \bigl (\gamma ({\overline{z}})^*\bigr )^{(m)}, \end{aligned} \end{aligned}$$

and one has $\overline{M(z)}^{-1} (\gamma ({\overline{z}})^*)^{(q)}\in {\mathfrak S}_r\big (L^2(\Omega ),L^2(\partial \Omega )\big )$ for $r> (n-1)/[2q+(3/2)]$ by (5.37) and each summand (and hence also the finite sum) on the right-hand side is in ${\mathfrak S}_r\big (L^2(\Omega ),L^2(\partial \Omega )\big )$ for $r> (n-1)/[2p+1+2m+(3/2)] = (n-1)/[2q+(5/2)]$, which follows from (5.41) and (5.38). Hence one has

5.42 $$\begin{aligned} \bigl ( M(z)^{-1} \gamma ({\overline{z}})^*\bigr )^{(q)}&\in {\mathfrak S}_r\bigl (L^2(\Omega ),L^2(\partial \Omega )\bigr ) \end{aligned}$$

for $r > (n-1)/[2q+(3/2)]$ and $z \in \rho (A_{\beta })\cap \rho (A_{\beta _1}).$ From (5.38) and (5.42) one then concludes that

$$\begin{aligned} \overline{\gamma (z)}^{(p)}\bigl ( M(z)^{-1} \gamma ({\overline{z}})^*\bigr )^{(q)}&\in {\mathfrak S}_r\bigl (L^2(\Omega )\bigr ) \end{aligned}$$

for $r > (n-1)/[2(p+q)+3] = (n-1)/(4k+3)$ and since $k\ge (n-3)/4$, one has $1> (n-1)/(4k+3)$, that is, the trace class condition (5.32) is satisfied. The same argument shows that (5.33) is satisfied. Finally, (5.34) follows from (5.40) and the fact that $k\ge (n-3)/4$ implies

$$\begin{aligned} \frac{2k+1}{j}\ge \frac{n-1}{2j}>\frac{n-1}{2j+1},\quad j=1,\dots ,2k+1. \end{aligned}$$

Hence the assumptions in Theorem 4.1 are satisfied with *S* in (5.15), the quasi boundary triple in (5.17) and the corresponding $\gamma$-field and Weyl function in (5.19) and (5.20), respectively. Now Theorem 4.1 yields assertion (*i*) in Theorem 5.3 with $A_{\beta _0}$ replaced by $A_\beta$ and for any orthonormal basis $(\varphi _j)_{j \in J}$ in $L^2(\partial \Omega )$ the function

$$\begin{aligned} \xi _1(\lambda ) =\sum _{j \in J} \lim _{\varepsilon \downarrow 0}\pi ^{-1} \bigl (\mathrm {Im}\bigl ( \log ({\mathcal {M}}_1(\lambda +i\varepsilon )) \bigr ) \varphi _j,\varphi _j\bigr )_{L^2(\partial \Omega )} \quad \text { for a.e.}~\lambda \in {\mathbb {R}}, \end{aligned}$$

is a spectral shift function for the pair $\{A_\beta ,A_{\beta _1}\}$ such that $\xi _1(\lambda )=0$ for $\lambda < \min (\sigma (A_\beta ))\le \min (\sigma (A_{\beta _1}))$ and the trace formula

$$\begin{aligned} {{\mathrm{tr}}}_{L^2(\Omega )}\left( (A_{\beta _1} - z I_{L^2(\Omega )})^{-(2k+1)} - (A_\beta - z I_{L^2(\Omega )})^{-(2k+1)}\right) = - (2k+1) \int _{\mathbb {R}}\frac{\,\xi _1(\lambda )\, d\lambda }{(\lambda - z)^{2k+2}} \end{aligned}$$

is valid for all $z \in \rho (A_\beta )\cap \rho (A_{\beta _1})$.

The same construction as above with $\beta _1$ replaced by $\beta _0$ yields an analogous representation for a spectral shift function $\xi _0$ of the pair $\{A_\beta ,A_{\beta _0}\}$. Finally it follows from the considerations in the end of Sect. 4 (see (4.29)) that

$$\begin{aligned} \begin{aligned} \xi (\lambda )&=\xi _1(\lambda )-\xi _0(\lambda )\\&=\sum _{j \in J} \lim _{\varepsilon \downarrow 0}\pi ^{-1} \Bigl (\bigl (\mathrm {Im}\bigl ( \log ({\mathcal {M}}_1(\lambda +i\varepsilon )) - \log ({\mathcal {M}}_0(\lambda + i \varepsilon ))\bigr )\bigr ) \varphi _j,\varphi _j\Bigr )_{L^2(\partial \Omega )} \end{aligned} \end{aligned}$$

for a.e. $\lambda \in {\mathbb {R}}$ is a spectral shift function for the pair $\{A_{\beta _0},A_{\beta _1}\}$ such that $\xi (\lambda )=0$ for $\lambda < \min (\sigma (A_{\beta }))\le \min (\sigma (A_{\beta _p}))$, $p=0,1$. This completes the proof of Theorem 5.3. $\square$

In space dimensions $n=2$ and $n=3$ one can choose $k=0$ in Theorem 5.3, and hence the resolvent difference of $A_{\beta _1}$ and $A_{\beta _0}$ is a trace class operator. In this situation Corollary 4.2 leads to the following slightly stronger statement.

### Corollary 5.4

Let the assumptions be as in Theorem 5.3 and suppose that $n=2$ or $n=3$. Then the following assertions (*i*)–(*iii*) hold:

(i) The difference of the resolvents of $A_{\beta _1}$ and $A_{\beta _0}$ is a trace class operator, that is,
  $$\begin{aligned} \big [(A_{\beta _1} - z I_{L^2(\Omega )})^{-1}-(A_{\beta _0} - z I_{L^2(\Omega )})^{-1}\big ] \in {\mathfrak S}_1\bigl (L^2(\Omega )\bigr ) \end{aligned}$$
  holds for all $z\in \rho (A_{\beta _1})\cap \rho (A_{\beta _0})$.
(ii) $\mathrm {Im}(\log ({\mathcal {M}}_p(z)))\in {\mathfrak S}_1(L^2(\partial \Omega ))$ for all $z\in {\mathbb {C}}\backslash {\mathbb {R}}$ and $p=0,1$, and the limit
  $$\begin{aligned} \mathrm {Im}\bigl (\log ({\mathcal {M}}_p(\lambda +i 0))\bigr ):=\lim _{\varepsilon \downarrow 0}\mathrm {Im}\bigl (\log ({\mathcal {M}}_p(\lambda +i\varepsilon ))\bigr ) \end{aligned}$$
  exists for a.e. $\lambda \in {\mathbb {R}}$ and $p=0,1$ in ${\mathfrak S}_1(L^2(\partial \Omega ))$.
(iii) The function
  $$\begin{aligned} \xi (\lambda )=\pi ^{-1} {{\mathrm{tr}}}_{L^2(\partial \Omega )}\bigl (\mathrm {Im}\big (\log ({\mathcal {M}}_1(\lambda + i0))-\log ({\mathcal {M}}_0(\lambda + i0)) \big )\bigr ) \quad \text { for a.e.}~\lambda \in {\mathbb {R}}, \end{aligned}$$
  is a spectral shift function for the pair $\{A_{\beta _0},A_{\beta _1}\}$ such that $\xi (\lambda )=0$ for $\lambda < \min (\sigma (A_\beta ))$ and the trace formula
  $$\begin{aligned} {{\mathrm{tr}}}_{L^2(\Omega )}\bigl ( (A_{\beta _1} - z I_{L^2(\Omega )})^{-1} - (A_{\beta _0} - z I_{L^2(\Omega )})^{-1}\bigr ) = - (2k+1) \int _{\mathbb {R}}\frac{\,\xi (\lambda )\, d\lambda }{(\lambda - z)^2} \end{aligned}$$
  is valid for all $z \in \rho (A_{\beta _0})\cap \rho (A_{\beta _1})$.

## Schrödinger operators with compactly supported potentials

In this section we determine a spectral shift function for the self-adjoint operators $\{-\Delta ,-\Delta +V\}$ in $L^2({\mathbb {R}}^n)$, $n \in {\mathbb {N}}$, $n\ge 2$, where it is assumed that $V\in L^\infty ({\mathbb {R}}^n)$ is a compactly supported real-valued function. Thus we consider the self-adjoint operators

6.1 $$\begin{aligned} A=-\Delta ,\quad B=-\Delta +V,\qquad {{\mathrm{dom}}}(A)={{\mathrm{dom}}}(B)=H^2({\mathbb {R}}^n), \end{aligned}$$

in $L^2({\mathbb {R}}^n)$, and we fix an open ball ${\mathcal {B}}_+\subset {\mathbb {R}}^n$ such that $\text {supp}\, (V) \subset {\mathcal {B}}_+$. The $n-1$ dimensional sphere $\partial {\mathcal {B}}_+$ is denoted by ${\mathcal {S}}$. We shall also make use of the self-adjoint Dirichlet realizations

6.2 $$\begin{aligned} A_+=-\Delta , \quad B_+=-\Delta +V,\quad {{\mathrm{dom}}}(A_+)={{\mathrm{dom}}}(B_+)=H^2({\mathcal {B}}_+)\cap H^1_0({\mathcal {B}}_+), \end{aligned}$$

of $-\Delta$ and $-\Delta +V$ in $L^2({\mathcal {B}}_+)$. Their spectra are discrete and bounded from below. The eigenvalue counting functions are denoted by $N(\, \cdot \,,A_+)$ and $N(\, \cdot \,,B_+)$, respectively; recall that $N(\lambda ,A_+)$ and $N(\lambda ,B_+)$ stand for the total number of eigenvalues (multiplicities counted) of $A_+$ and $B_+$ in $(-\infty , \lambda )$, $\lambda \in {\mathbb {R}}$.

The main ingredient in the proof of Theorem  6.1 below is a decoupling technique for the operators *A* and *B*, where artificial Dirichlet boundary conditions on the sphere ${\mathcal {S}}$ will be imposed. We shall use the extension of the $L^2({\mathcal {S}})$ scalar product onto the dual pair $H^{1/2}({\mathcal {S}})\times H^{-1/2}({\mathcal {S}})$ via

6.3 $$\begin{aligned} \langle \varphi ,\psi \rangle =\bigl (\imath \varphi ,\widetilde{\imath ^{-1}}\psi \bigr )_{L^2({\mathcal {S}})},\quad \varphi \in H^{1/2}({\mathcal {S}}),\quad \psi \in H^{-1/2}({\mathcal {S}}), \end{aligned}$$

where $\imath$ is a uniformly positive self-adjoint operator in $L^2({\mathcal {S}})$ defined on the dense subspace $H^{1/2}({\mathcal {S}})$ (and in the following $\iota$ is regarded as an isomorphism from $H^{1/2}({\mathcal {S}})$ onto $L^2({\mathcal {S}})$), and $\widetilde{\imath ^{-1}}$ is the extension of $\imath ^{-1}$ to an isomorphism from $H^{-1/2}({\mathcal {S}})$ onto $L^2({\mathcal {S}})$. A typical and convenient choice for $\imath$ is $(-\Delta _{\mathcal {S}}+I_{L^2({\mathcal {S}})})^{1/4}$, where $-\Delta _{\mathcal {S}}$ is the Laplace–Beltrami operator on the sphere ${\mathcal {S}}$; for other choices see also [8, Remark 5.3].

Since $\langle \cdot ,\cdot \rangle$ in (6.3) is an extension of the $L^2({\mathcal {S}})$ scalar product, Green’s identity can also be written in the form

6.4 $$\begin{aligned} (-\Delta f_+,g_+)_{L^2({\mathcal {B}}_+)}-(f_+,-\Delta g_+)_{L^2({\mathcal {B}}_+)}=\langle \gamma _D^+ f_+,\gamma _N^+ g_+ \rangle -\langle \gamma _N^+ f_+,\gamma _D^+ g_+\rangle \qquad \end{aligned}$$

for $f_+,g_+\in H^2({\mathcal {B}}_+)$. Here $\gamma _D^+$ and $\gamma _N^+$ denote the Dirichlet and Neumann trace operators in (5.3) (with $\Omega$ and $\partial \Omega$ replaced by ${\mathcal {B}}_+$ and ${\mathcal {S}}$, respectively). Let ${\mathcal {B}}_-:={\mathbb {R}}^n\backslash \overline{{\mathcal {B}}}_+$ and let $\gamma _D^-$ and $\gamma _N^-$ be the Dirichlet and Neumann trace operators on ${\mathcal {B}}_-$; the normal vector in the definition of $\gamma _N^-$ is pointing in the outward direction of ${\mathcal {B}}_-$ and hence opposite to the normal of ${\mathcal {B}}_+$. Besides (6.4) we also have the corresponding Green’s identity on ${\mathcal {B}}_-$, that is,

6.5 $$\begin{aligned} (-\Delta f_-,g_-)_{L^2({\mathcal {B}}_-)}-(f_-,-\Delta g_-)_{L^2({\mathcal {B}}_-)}=\langle \gamma _D^- f_-,\gamma _N^- g_- \rangle -\langle \gamma _N^- f_-,\gamma _D^- g_-\rangle \nonumber \\ \end{aligned}$$

holds for all $f_-,g_-\in H^2({\mathcal {B}}_-)$.

Next we define Dirichlet-to-Neumann maps associated to $-\Delta$ and $-\Delta +V$ on ${\mathcal {B}}_+$ and $-\Delta$ on ${\mathcal {B}}_-$ as operators from $H^{1/2}({\mathcal {S}})$ to $H^{-1/2}({\mathcal {S}})$. First, we recall that for $z \not \in \sigma (A_+)$ and $\varphi \in H^{1/2}({\mathcal {S}})$ there exists a unique solution $f_z \in H^1({\mathcal {B}}_+)$ of the boundary value problem $-\Delta f_z = z f_z$, $\gamma _D^+ f_z =\varphi$. The corresponding solution operator is $P_+(z):H^{1/2}({\mathcal {S}})\rightarrow H^1({\mathcal {B}}_+)$, $\varphi \mapsto f_z$, and for $z \not \in \sigma (A_+)$, the *Dirichlet-to-Neumann map* ${\mathcal {D}}_+(z)$ *associated to* $-\Delta$ *in* ${\mathcal {B}}_+$ is defined by

$$\begin{aligned} {\mathcal {D}}_+(z):H^{1/2}({\mathcal {S}})\rightarrow H^{-1/2}({\mathcal {S}}),\quad \varphi \mapsto \gamma _N^+ P_+(z)\varphi . \end{aligned}$$

Similarly, for $\zeta \not \in \sigma (B_+)$ and $\psi \in H^{1/2}({\mathcal {S}})$, there exists a unique solution $g_\zeta \in H^1({\mathcal {B}}_+)$ of the boundary value problem $(-\Delta +V)g_\zeta = \zeta g_\zeta$ , $\gamma _D^+ g_\zeta = \psi$. The corresponding solution operator is $P_+^V(\zeta ):H^{1/2}({\mathcal {S}})\rightarrow H^1({\mathcal {B}}_+)$, $\psi \mapsto g_\zeta$, and for $\zeta \not \in \sigma (B_+)$ the *Dirichlet-to-Neumann map* ${\mathcal {D}}_+^V(\zeta )$ *associated to* $-\Delta +V$ *in* ${\mathcal {B}}_+$ is defined by

$$\begin{aligned} {\mathcal {D}}_+^V(\zeta ):H^{1/2}({\mathcal {S}})\rightarrow H^{-1/2}({\mathcal {S}}),\quad \psi \mapsto \gamma _N^+ P_+^V(\zeta )\psi . \end{aligned}$$

Furthermore, for $\zeta ' \not \in [0,\infty )$ and $\xi \in H^{1/2}({\mathcal {S}})$ there exists a unique solution $h_{\zeta '}\in H^1({\mathcal {B}}_-)$ of the boundary value problem $-\Delta h_{\zeta '} = \zeta ' h_{\zeta '}$, $\gamma _D^- h_{\zeta '}=\xi$. As above the solution operator is $P_-(\zeta '):H^{1/2}({\mathcal {S}})\rightarrow H^1({\mathcal {B}}_-)$, $\xi \mapsto h_{\zeta '}$, and for $\zeta ' \not \in [0,\infty )$, the *Dirichlet-to-Neumann map* ${\mathcal {D}}_-(\zeta ')$ *associated to* $-\Delta$ *in* ${\mathcal {B}}_-$ is defined by

$$\begin{aligned} {\mathcal {D}}_-(\zeta '):H^{1/2}({\mathcal {S}})\rightarrow H^{-1/2}({\mathcal {S}}),\quad \xi \mapsto \gamma _N^- P_-(\zeta ')\xi . \end{aligned}$$

One recalls that the Dirichlet-to-Neumann maps ${\mathcal {D}}_+(z)$, ${\mathcal {D}}_+^V(\zeta )$, and ${\mathcal {D}}_-(\zeta ')$ above are bounded operators from $H^{1/2}({\mathcal {S}})$ to $H^{-1/2}({\mathcal {S}})$. Moreover, for $z\in {\mathbb {C}}\backslash {\mathbb {R}}$, each of the Dirichlet-to-Neumann maps is boundedly invertible and the same is true for the sums

$$\begin{aligned} \begin{aligned} {\mathcal {D}}_+(z)+ {\mathcal {D}}_-(z):H^{1/2}({\mathcal {S}})\rightarrow H^{-1/2}({\mathcal {S}})&,\quad z\in {\mathbb {C}}\backslash {\mathbb {R}},\\ {\mathcal {D}}_+^V(z)+ {\mathcal {D}}_-(z):H^{1/2}({\mathcal {S}})\rightarrow H^{-1/2}({\mathcal {S}})&,\quad z\in {\mathbb {C}}\backslash {\mathbb {R}}. \end{aligned} \end{aligned}$$

Hence, the operators

6.6 $$\begin{aligned} \begin{aligned} {\mathfrak N}(z)&=\imath \bigl ({\mathcal {D}}_+(z)+ {\mathcal {D}}_-(z)\bigr )^{-1}\,\widetilde{\imath }:L^2({\mathcal {S}})\rightarrow L^2({\mathcal {S}}), \quad z\in {\mathbb {C}}\backslash {\mathbb {R}},\\ {\mathfrak N}_V(z)&=\imath \bigl ({\mathcal {D}}_+^V(z)+ {\mathcal {D}}_-(z)\bigr )^{-1}\,\widetilde{\imath }:L^2({\mathcal {S}})\rightarrow L^2({\mathcal {S}}), \quad z\in {\mathbb {C}}\backslash {\mathbb {R}}, \end{aligned} \end{aligned}$$

are everywhere defined and bounded in $L^2({\mathcal {S}})$.

In the next theorem we obtain a representation for a spectral shift function for $\{A,B\}$ in (6.1) via a decoupling technique and Theorem 4.1. The considerations in the beginning of Step 1 of the proof of Theorem 6.1 are similar as in [8, Section 5.2] and hence some details are omitted.

### Theorem 6.1

Let $n \in {\mathbb {N}}$, $n\ge 2$, and $k \in {\mathbb {N}}$, $k> (n-2)/4$, and suppose that $V\in L^\infty ({\mathbb {R}}^n)$ is real-valued with support in the open ball ${\mathcal {B}}_+$. In addition, let ${\mathfrak N}(z)$ and ${\mathfrak N}_V(z)$ be as in (6.6), and denote the eigenvalue counting functions of the Dirichlet operators $A_+$ and $B_+$ in $L^2({\mathcal {B}}_+)$ by $N(\, \cdot \,,A_+)$ and $N(\, \cdot \,,B_+)$, respectively. Then the following assertions (*i*) and (*ii*) hold:

(i) The difference of the $2k+1$th-powers of the resolvents of *A* and *B* is a trace class operator, that is,
  $$\begin{aligned} \left[ \left( B - z I_{L^2({\mathbb {R}}^n)}\right) ^{-(2k+1)}-\left( A - z I_{L^2({\mathbb {R}}^n)}\right) ^{-(2k+1)}\right] \in {\mathfrak S}_1\bigl (L^2({\mathbb {R}}^n)\bigr ) \end{aligned}$$
  holds for all $z \in \rho (B)=\rho (A)\cap \rho (B)$.
(ii) For any orthonormal basis $(\varphi _j)_{j \in J}$ in $L^2({\mathcal {S}})$ the function
  $$\begin{aligned} \xi (\lambda )&=\sum _{j \in J} \lim _{\varepsilon \downarrow 0}\pi ^{-1} \Bigl (\bigl (\mathrm {Im}\bigl ( \log ({\mathfrak N}(\lambda +i\varepsilon )) - \log ({\mathfrak N}_V(\lambda + i \varepsilon ))\bigr )\bigr ) \varphi _j,\varphi _j\Bigr )_{L^2({\mathcal {S}})} \\&\quad + N(\lambda ,B_+) - N(\lambda ,A_+)\quad \text { for a.e. } \lambda \in {\mathbb {R}}, \end{aligned}$$
  is a spectral shift function for the pair $\{A,B\}$ such that $\xi (\lambda )=0$ for $\lambda < \min (\sigma (B))\le 0$ and the trace formula
  $$\begin{aligned} {{\mathrm{tr}}}_{L^2({\mathbb {R}}^n)}\left( \left( B - z I_{L^2({\mathbb {R}}^n)}\right) ^{-(2k+1)}-\left( A - z I_{L^2({\mathbb {R}}^n)}\right) ^{-(2k+1)}\right) = - (2k+1) \int _{\mathbb {R}}\frac{\xi (\lambda )\, d\lambda }{(\lambda - z)^{2k+2}} \end{aligned}$$
  is valid for all $z \in \rho (B)=\rho (A)\cap \rho (B)$.

### Proof

Besides the self-adjoint operators $A=-\Delta$ and $B=-\Delta +V$ in (6.1), and the Dirichlet realizations $A_+=-\Delta$ and $B_+=-\Delta +V$ in $L^2({\mathcal {B}}_+)$ in (6.2) we shall also make use of the Dirichlet realization $A_-$ of $-\Delta$ in $L^2({\mathcal {B}}_-)$ given by

6.7 $$\begin{aligned} A_-=-\Delta ,\quad {{\mathrm{dom}}}(A_-)=H^2({\mathcal {B}}_-)\cap H^1_0({\mathcal {B}}_-), \end{aligned}$$

as well as the orthogonal sums in $L^2({\mathbb {R}}^n)=L^2({\mathcal {B}}_+)\oplus L^2({\mathcal {B}}_-)$,

6.8 $$\begin{aligned} \begin{aligned}&A_D:=\begin{pmatrix} A_+ &{} 0 \\ 0 &{} A_-\end{pmatrix}\quad \text { and } \quad B_D:=\begin{pmatrix} B_+ &{} 0 \\ 0 &{} A_-\end{pmatrix}, \\&{{\mathrm{dom}}}(A_D)={{\mathrm{dom}}}(B_D)=\bigl (H^2({\mathcal {B}}_+)\cap H^1_0({\mathcal {B}}_+)\bigr )\times \bigl (H^2({\mathcal {B}}_-)\cap H^1_0({\mathcal {B}}_-)\bigr ). \end{aligned} \end{aligned}$$

For any orthonormal basis $(\varphi _j)_{j \in J}$ in $L^2({\mathcal {S}})$ we shall first prove the representation

6.9 $$\begin{aligned} \xi _A(\lambda )=\sum _{j \in J} \lim _{\varepsilon \downarrow 0} \pi ^{-1} \bigl (\mathrm {Im}\bigl (\log ({\mathfrak N}(\lambda + i\varepsilon ))\bigr )\varphi _j,\varphi _j\bigr )_{L^2({\mathcal {S}})} \end{aligned}$$

for a spectral shift function $\xi _A$ of the pair $\{A,A_D\}$ and the representation

6.10 $$\begin{aligned} \xi _B(\lambda )=\sum _{j \in J} \lim _{\varepsilon \downarrow 0} \pi ^{-1} \bigl (\mathrm {Im}\bigl (\log ({\mathfrak N}_V(\lambda + i\varepsilon ))\bigr )\varphi _j,\varphi _j\bigr )_{L^2({\mathcal {S}})} \end{aligned}$$

for a spectral shift function $\xi _B$ of the pair $\{B,B_D\}$.

*Step 1*  In this step we consider the operators *B* and $B_D$ as self-adjoint extensions of the closed symmetric $S=B\cap B_D$, which is given by

6.11 $$\begin{aligned} S = - \Delta +V, \quad {{\mathrm{dom}}}(S) = \bigl \{f\in H^2({\mathbb {R}}^n) \, \big | \, \gamma _D^+f_+=0=\gamma _D^-f_-\bigr \}. \end{aligned}$$

Furthermore, consider the operator

$$\begin{aligned} T=-\Delta +V, \quad {{\mathrm{dom}}}(T) = \left\{ f=\begin{pmatrix} f_+\\ f_-\end{pmatrix}\in H^2({\mathcal {B}}_+)\times H^2({\mathcal {B}}_-) \, \bigg | \, \gamma _D^+f_+=\gamma _D^-f_-\right\} , \end{aligned}$$

and set $\gamma _D f:= \gamma _D^+f_+=\gamma _D^-f_-$ for $f\in {{\mathrm{dom}}}(T)$. It is easy to see with the help of Theorem 2.2, (6.4)–(6.5) and (5.3) that $\{L^2({\mathcal {S}}),\Gamma _0,\Gamma _1\}$, where

6.12 $$\begin{aligned} \Gamma _0 f=\widetilde{\imath ^{-1}}(\gamma _N^+f_++\gamma _N^-f_-) \quad \text { and } \quad \Gamma _1 f=\imath \gamma _D f,\quad f\in {{\mathrm{dom}}}(T), \end{aligned}$$

is a quasi boundary triple for $T\subset S^*$ and $B=T\upharpoonright \ker (\Gamma _0)$ and $B_D=T\upharpoonright \ker (\Gamma _1)$ hold (cf. the proof of [8, Theorem 5.1]). The corresponding Weyl function is

6.13 $$\begin{aligned} M(z)\varphi =\imath \bigl ({\mathcal {D}}_+^V(z)+ {\mathcal {D}}_-(z)\bigr )^{-1}\widetilde{\imath }\varphi = {\mathfrak N}_V(z)\varphi \end{aligned}$$

for all $z \in \rho (B)\cap \rho (B_D)$ and $\varphi \in {{\mathrm{ran}}}(\Gamma _0)$. Furthermore, the proof of [8, Theorem 5.1] shows that *M*(*z*) and $M(z)^{-1}$ are bounded for all $z \in \rho (B)\cap \rho (B_D)$ and one has $\overline{M(z)}={\mathfrak N}_V(z)$.

One observes that *B* corresponds to the densely defined, closed quadratic form

$$\begin{aligned} \mathfrak b[f,g]=(\nabla f,\nabla g)_{(L^2({\mathbb {R}}^n))^n}+(Vf,g)_{L^2({\mathbb {R}}^n)},\quad {{\mathrm{dom}}}(\mathfrak b)=H^1({\mathbb {R}}^n), \end{aligned}$$

and that $B_D$ corresponds to the densely defined closed quadratic form

$$\begin{aligned} \mathfrak b_D[f,g]=(\nabla f,\nabla g)_{(L^2({\mathbb {R}}^n))^n}+(Vf,g)_{L^2({\mathbb {R}}^n)},\quad {{\mathrm{dom}}}(\mathfrak b_D)=H^1_0({\mathcal {B}}_+)\times H^1_0({\mathcal {B}}_-). \end{aligned}$$

Since $H^1({\mathbb {R}}^n)\subset (H^1_0({\mathcal {B}}_+)\times H^1_0({\mathcal {B}}_-))$ this implies $\mathfrak b\le \mathfrak b_D$ and yields the sign condition $(B - \zeta I_{L^2({\mathbb {R}}^n)})^{-1}\ge (B_D - \zeta I_{L^2({\mathbb {R}}^n)})^{-1}$ in the assumptions of Theorem 4.1 for all $\zeta < \min (\sigma (B))\le \min (\sigma (B_D))$; see the beginning of Step 2 in the proof of Theorem 5.3.

Next, we verify the ${\mathfrak S}_p$-conditions

6.14 $$\begin{aligned} \, \overline{\gamma (z)}^{(p)}\bigl ( M(z)^{-1} \gamma ({\overline{z}})^*\bigr )^{(q)}\in & {} {\mathfrak S}_1\bigl (L^2({\mathbb {R}}^n)\bigr ),\quad p+q=2k, \end{aligned}$$

6.15 $$\begin{aligned} \bigl ( M(z)^{-1} \gamma ({\overline{z}})^*\bigr )^{(q)}\overline{\gamma (z)}^{(p)}\in & {} {\mathfrak S}_1\bigl (L^2({\mathcal {S}})\bigr ),\quad p+q=2k, \end{aligned}$$

and

6.16 $$\begin{aligned} \frac{d^j}{dz^j} \overline{M (z)}\in {\mathfrak S}_{(2k+1)/j}\bigl (L^2({\mathcal {S}})\bigr ),\quad j=1,\dots ,2k+1, \end{aligned}$$

for all $z \in \rho (B)\cap \rho (B_D)$ in the assumptions of Theorem 4.1. For this we use the smoothing property

6.17 $$\begin{aligned} \left( B - z I_{L^2({\mathbb {R}}^n)}\right) ^{-1}f \in H^{\ell +2}_{\mathcal {O}}({\mathbb {R}}^n)\quad \text { for all } \, f\in H^{\ell }_{\mathcal {O}}({\mathbb {R}}^n) \quad \text { and }\quad \ell \in {\mathbb {N}}_0, \end{aligned}$$

where ${\mathcal {O}}$ is an open neighborhood of the sphere ${\mathcal {S}}$ in ${\mathbb {R}}^n$ such that $\text {supp}\, (V) \cap {\mathcal {O}}=\emptyset$ and $H^{\ell }_{\mathcal {O}}({\mathbb {R}}^n) = \big \{f\in L^2({\mathbb {R}}^n) \, \big | \, f\upharpoonright _{\mathcal {O}}\in H^{\ell }({\mathcal {O}})\big \}$ (cf. [5, Lemma 4.1(i)]).

It follows from (2.2) and the definition of $\Gamma _1$ that

6.18 $$\begin{aligned} \gamma ({\overline{z}})^* f=\Gamma _1\left( B - z I_{L^2({\mathbb {R}}^n)}\right) ^{-1}f = \imath \, \gamma _D \left( B - z I_{L^2({\mathbb {R}}^n)}\right) ^{-1}f \end{aligned}$$

and hence (2.6) yields

6.19 $$\begin{aligned} \bigl (\gamma ({\overline{z}})^*\bigr )^{(q)} = q! \, \gamma ({\overline{z}})^*\left( B - z I_{L^2({\mathbb {R}}^n)}\right) ^{-q} = q! \, \imath \, \gamma _D\left( B - z I_{L^2({\mathbb {R}}^n)}\right) ^{-(q+1)}. \end{aligned}$$

Since

$$\begin{aligned} {{\mathrm{ran}}}\left( \gamma _D\left( B - z I_{L^2({\mathbb {R}}^n)}\right) ^{-(q+1)}\right) \subset H^{2q+(3/2)}({\mathcal {S}}) \end{aligned}$$

by (6.17) and (5.5), and the operator $\gamma _D(B - z I_{L^2({\mathbb {R}}^n)})^{-(q+1)}$ is bounded from $L^2({\mathbb {R}}^n)$ into $H^{1/2}({\mathcal {S}})$ it follows from Lemma 5.2 with $s=2q+(3/2)$ and $t=1/2$ that

$$\begin{aligned} \gamma _D\left( B - z I_{L^2({\mathbb {R}}^n)}\right) ^{-(q+1)}\in {\mathfrak S}_r\bigl (L^2({\mathbb {R}}^n),H^{1/2}({\mathcal {S}})\bigr ). \quad r> (n-1)/(2q+1), \end{aligned}$$

As $\imath :H^{1/2}({\mathcal {S}})\rightarrow L^2({\mathcal {S}})$ is an isomorphism one concludes from (6.19) that

6.20 $$\begin{aligned} \left( \gamma ({\overline{z}})^*\right) ^{(q)} \in {\mathfrak S}_r\bigl (L^2({\mathbb {R}}^n),L^2({\mathcal {S}})\bigr ), \quad r> (n-1)/(2q+1), \end{aligned}$$

for all $z \in \rho (B)$ and $q \in {\mathbb {N}}_0$. From this it is also clear that

6.21 $$\begin{aligned} \overline{\gamma (z)}^{(p)}\in {\mathfrak S}_r\bigl (L^2({\mathcal {S}}),L^2({\mathbb {R}}^n)\bigr ), \quad r> (n-1)/(2p+1), \end{aligned}$$

for all $z \in \rho (B)$ and $p \in {\mathbb {N}}_0$. Furthermore,

6.22 $$\begin{aligned} \frac{d^j}{dz^j} \overline{M(z)}=j! \, \gamma ({\overline{z}})^*\left( B - z I_{L^2({\mathbb {R}}^n)}\right) ^{-(j-1)}\overline{\gamma (z)}, \quad j \in {\mathbb {N}}, \end{aligned}$$

by (2.12), and using (6.18) one obtains with the arguments above that

$$\begin{aligned} \gamma ({\overline{z}})^*\left( B - z I_{L^2({\mathbb {R}}^n)}\right) ^{-(j-1)} = \imath \gamma _D \left( B - z I_{L^2({\mathbb {R}}^n)}\right) ^{-j} \in {\mathfrak S}_r\bigl (L^2({\mathbb {R}}^n),L^2({\mathcal {S}})\bigr ) \end{aligned}$$

for $r> (n-1)/(2j-1),$ $z \in \rho (B)$, and $j \in {\mathbb {N}}$. Together with (6.21) for $p=0$ one finds that (6.22) satisfies

6.23 $$\begin{aligned} \frac{d^j}{dz^j} \overline{M (z)} \in {\mathfrak S}_r\bigl (L^2({\mathcal {S}})\bigr ), \quad r> (n-1)/(2j), \end{aligned}$$

for all $z \in \rho (B)$ and $j \in {\mathbb {N}}$. The same arguments as in Step 3 of the proof Theorem 5.3 show that

6.24 $$\begin{aligned} \frac{d^j}{dz^j} \overline{M (z)}^{-1}\in {\mathfrak S}_r\bigl (L^2({\mathcal {S}})\bigr ), \quad r> (n-1)/(2j), \end{aligned}$$

for all $z \in \rho (B)\cap \rho (B_D)$ and $j \in {\mathbb {N}}$. It follows from (6.20) and (6.24) that each summand in the right-hand side in

$$\begin{aligned} \bigl ( M(z)^{-1} \gamma ({\overline{z}})^* \bigr )^{(q)}= \sum _{\begin{array}{c} p+m=q \\ p,m\ge 0 \end{array}} \begin{pmatrix} q \\ p \end{pmatrix} \bigl (\overline{M(z)}^{-1}\bigr )^{(p)} \bigl (\gamma ({\overline{z}})^*\bigr )^{(m)} \end{aligned}$$

belongs to ${\mathfrak S}_r\big (L^2({\mathcal {S}}),L^2({\mathbb {R}}^n)\big )$ for $r> (n-1)/(2q+1)$, and hence one infers together with (6.21) that

$$\begin{aligned} \overline{\gamma (z)}^{(p)}\bigl ( M(z)^{-1} \gamma ({\overline{z}})^*\bigr )^{(q)}&\in {\mathfrak S}_r\bigl (L^2({\mathbb {R}}^n)\bigr ) \end{aligned}$$

for $r> (n-1)/[2(p+q)+2]= (n-1)/(4k+2),$ and since $k> (n-2)/4$ by assumption, one has $1> (n-1)/(4k+2)$, implying the trace class condition (6.14). The same argument shows that (6.15) is satisfied. Finally, (6.16) follows from (6.23) and the fact that $k> (n-2)/4$ implies

$$\begin{aligned} \frac{2k+1}{j}>\frac{n}{2j}>\frac{n-1}{2j}, \quad j=1,\dots ,2k+1. \end{aligned}$$

Hence, the assumptions in Theorem 4.1 are satisfied with *S* in (6.11), the quasi boundary triple in (6.12), and the corresponding Weyl function in (6.13). Thus, Theorem 4.1 yields assertion (*i*) with *A* replaced by $B_D$ and for any orthonormal basis $(\varphi _j)_{j \in J}$ in $L^2({\mathcal {S}})$ the function

$$\begin{aligned} \xi _B(\lambda ) =\sum _{j \in J} \lim _{\varepsilon \downarrow 0}\pi ^{-1} \bigl (\mathrm {Im}\bigl ( \log ({\mathfrak N}_V(\lambda +i\varepsilon )) \bigr ) \varphi _j,\varphi _j\bigr )_{L^2({\mathcal {S}})} \quad \text { for a.e.}~\lambda \in {\mathbb {R}}\end{aligned}$$

in (6.10) is a spectral shift function for the pair $\{B,B_D\}$ and the trace formula

6.25 $$\begin{aligned} {{\mathrm{tr}}}_{L^2({\mathbb {R}}^n)}\left( \left( B_D - z I_{L^2({\mathbb {R}}^n)}\right) ^{-(2k+1)}-\left( B - z I_{L^2({\mathbb {R}}^n)}\right) ^{-(2k+1)}\right) = - (2k+1) \int _{\mathbb {R}}\frac{\xi _B(\lambda )\, d\lambda }{(\lambda - z)^{2k+2}}\nonumber \\ \end{aligned}$$

is valid for all $z \in \rho (B)\cap \rho (B_D)$.

*Step 2*  Now we complete the proof of Theorem 6.1. First, we note that the same arguments as in Step 1 with $V=0$ show that assertion (*i*) in Theorem 6.1 holds with *B* replaced by $A_D$ and $\xi _A$ in (6.9) is a spectral shift function for the pair $\{A,A_D\}$ such that

6.26 $$\begin{aligned} {{\mathrm{tr}}}_{L^2({\mathbb {R}}^n)}\left( \left( A_D - z I_{L^2({\mathbb {R}}^n)}\right) ^{-(2k+1)}-\left( A - z I_{L^2({\mathbb {R}}^n)}\right) ^{-(2k+1)}\right) = - (2k+1) \int _{\mathbb {R}}\frac{\xi _A(\lambda )\, d\lambda }{(\lambda - z)^{2k+2}}\nonumber \\ \end{aligned}$$

holds for all $z \in {\mathbb {C}}\backslash [0,\infty )$. The assumption $k> (n-2)/4$ implies $2k+1> n/2$ and hence

6.27 $$\begin{aligned} \begin{aligned} (A_+ - z I_{L^2({\mathcal {B}}_+)})^{-(2k+1)}&\in {\mathfrak S}_1\bigl (L^2({\mathcal {B}}_+)\bigr ),\quad z\in \rho (A_+),\\ (B_+ - \zeta I_{L^2({\mathcal {B}}_+)})^{-(2k+1)}&\in {\mathfrak S}_1\bigl (L^2({\mathcal {B}}_+)\bigr ),\quad \zeta \in \rho (B_+), \end{aligned} \end{aligned}$$

by standard Weyl asymptotics. Furthermore, since the spectra of $A_+$ and $B_+$ are discrete and bounded from below, it is well-known that

6.28 $$\begin{aligned} \xi _+ (\lambda ) = N(\lambda ,B_+) - N(\lambda ,A_+), \quad \lambda \in {\mathbb {R}}, \end{aligned}$$

is a spectral shift function for the pair $\{A_+,B_+\}$ (see, e.g., [16, (3.28)]). From (6.8) it is clear that $\xi _+$ is also a spectral shift function for the pair $\{A_D,B_D\}$. Since

$$\begin{aligned} \big [(B_D - z I_{L^2({\mathbb {R}}^n)})^{-(2k+1)}-(A_D - z I_{L^2({\mathbb {R}}^n)})^{-(2k+1)}\big ] \in {\mathfrak S}_1\bigl (L^2({\mathbb {R}}^n)\bigr ) \end{aligned}$$

by (6.27) and (6.8) one concludes that

6.29 $$\begin{aligned} \begin{aligned} {{\mathrm{tr}}}_{L^2({\mathbb {R}}^n)}\bigl ( (B_D - z I_{L^2({\mathbb {R}}^n)})^{-(2k+1)}-(A_D - z I_{L^2({\mathbb {R}}^n)})^{-(2k+1)}\bigr ) = - (2k+1) \int _{\mathbb {R}}\frac{\xi _+(\lambda )\, d\lambda }{(\lambda - z)^{2k+2}} \end{aligned} \end{aligned}$$

for $z\in \rho (A_D) \cap \rho (B_D)$. Hence, $\xi (\lambda )=\xi _A(\lambda )-\xi _B(\lambda )+\xi _+(t)$ for a.e. $\lambda \in {\mathbb {R}}$ is a spectral shift function for the pair $\{A,B\}$, and taking into account the specific form of $\xi _A$, $\xi _B$, and $\xi _+$, in (6.9), (6.10), and (6.28) and the trace formulas (6.25), (6.26), and (6.29), the assertions in Theorem 6.1 follow. $\square$

### Remark 6.2

We note that the spectral shift function $\xi$ in Theorem 6.1 is continuous for $\lambda > 0$ since $V\in L^\infty ({\mathbb {R}}^n)$ is compactly supported (see, e.g., [76, Theorem 9.1.20]). On the other hand the spectral shift function $\xi _+$ of $\{A_+,B_+\}$ is a step function and hence the difference of the spectral shift functions $\xi _A$ and $\xi _B$ of the pairs $\{A,A_D\}$ and $\{B,B_D\}$ cancel the discontinuities of $\xi _+$ for $\lambda > 0$.
